# Impact of Newtonian heating and Fourier and Fick’s laws on a magnetohydrodynamic dusty Casson nanofluid flow with variable heat source/sink over a stretching cylinder

**DOI:** 10.1038/s41598-021-81747-x

**Published:** 2021-01-27

**Authors:** Muhammad Ramzan, Naila Shaheen, Jae Dong Chung, Seifedine Kadry, Yu-Ming Chu, Fares Howari

**Affiliations:** 1 Department of Computer Science, Bahria University, Islamabad, 44000 Pakistan; 2 Department of Mechanical Engineering, Sejong University, Seoul, 143-747 South Korea; 3 Department of Mathematics and Computer Science, Faculty of Science, Beirut Arab University, Beirut, 115020 Lebanon; 4 Department of Mathematics, Huzhou University, Huzhou, 313000 People’s Republic of China; 5 Hunan Provincial Key Laboratory of Mathematical Modeling and Analysis in Engineering, Changsha University of Science & Technology, Changsha, 410114 People’s Republic of China; 6 College of Natural and Health Sciences, Zayed University, 144543 Abu Dhabi, UAE

**Keywords:** Software, Mechanical engineering

## Abstract

The present investigation aims to deliberate the magnetohydrodynamic (MHD) dusty Casson nanofluid with variable heat source/sink and modified Fourier’s and Fick’s laws over a stretching cylinder. The novelty of the flow model is enhanced with additional effects of the Newtonian heating, activation energy, and an exothermic chemical reaction. In an exothermic chemical reaction, the energy of the reactants is higher than the end products. The solution to the formulated problem is attained numerically by employing the MATLAB software function bvp4c. The behavior of flow parameters versus involved profiles is discussed graphically at length. For large values of momentum dust particles, the velocity field for the fluid flow declines, whereas an opposite trend is perceived for the dust phase. An escalation is noticed for the Newtonian heating in the temperature profile for both the fluid and dust-particle phase. A comparison is also added with an already published work to check the validity of the envisioned problem.

## Introduction

Researchers have shown keen interest in the study of heat and mass transfer of fluid flow amalgamated with dust particles over a stretching surface due to its wide-ranging applications including wastewater treatment, cement production, environmental pollution, smoke emission from vehicle’s cooling effects of air conditioner, purification of crude oil, emission of effluents from industries and formation of raindrops. In a laminar flow, the impact of heat transfer on fluid flow with suspended particles is conducted by Saffman^1^. The flow of dusty Casson fluid with melting heat and Cattaneo Christov (CC) heat flux model past an extended sheet is numerically examined by Gireesha et al.^2^. In this study, it is understood that increment in the magnetic parameter and mass concentration parameter results in a decline of the velocity field for both phases. The influence of conjugate heat transfer with variable heat source/sink on a dusty Casson and Carreau fluid past a deforming sheet is studied by Mahanthesh et al.^3^. Bilal and Ramzan^4^ emphasized the nonlinear thermal radiation on a dusty nanofluid rotating flow with Hall current in a Darcy Forchheimer spongy medium. The main outcome of this investigation is that the rate of heat transfer escalates by amplifying the Prandtl number. Souayeh et al.^5^ numerically illustrated the outcome of heat transfer and radiation effect on hybrid nanofluid with dust particles on a stretching sheet. It is concluded that by increasing the thermal radiation more heat is transmuted to the fluid which results in enhancement of the temperature field. The influence of the (CC) heat flux model on nanofluid with the deferment of dust particles on an elongated cylinder is examined by Upadhya et al.^6^. Lately, researchers have pondered on the dusty fluid flows mentioned in Refs.^7–10^.

Non-Newtonian fluid flows over a stretching surface has immensely been emphasized by the researchers due to its vast applications such as cooling of nuclear reactors, production of glass fiber, manufacturing of electronic chips, the drilling process, and groundwater pollution, etc. Casson fluid is known as a shear-thinning fluid as it has distinct characteristics. Jelly, concentrated fruit juice, human blood, soup, tomato sauce, and honey are a few examples of Casson fluid. Naqvi et al.^11^ addressed the influence of thermal radiation on a magnetohydrodynamic Casson nanofluid flow on a stretching cylinder with Joule heating. Here, it is concluded that by increasing the curvature parameter, the velocity, temperature, and concentration profile escalate. The Casson nanofluid flow past a stretching cylinder with variable thermal conductivity and (CC) heat flux model in a porous medium is discussed by Tulu and Ibrahim^12^. It is concluded that for higher values of magnetic and permeability parameters, velocity field drops. Rehman et al.^13^ numerically illustrated the convective flow of an MHD Casson fluid with thermal stratification past a stretching cylindrical surface. It is noticed that the rate of heat transfer declines with an increase in Casson fluid parameter and exhibits an opposite behavior for curvature parameter. Ramesh et al.^14^ explored the outcome of the convective condition and thermal radiation on a dusty Casson fluid flow over a hollow stretching cylinder. Researchers have exhibited great interest in Casson fluid flow on an elongated surface which can be seen in Refs.^15–26^.

Newtonian heating plays a vital role in cooling and heating of buildings, heat exchanger designing, conjugate heat transfer around fins, petroleum industry and solar radiation, etc. Four discrete heat transfer types from the surface to the ambient liquid are defined by Merkin^27^. Casson fluid flow with dust particles past a vertical deforming sheet with a modified magnetic field and conjugate heat transfer is deliberated by Kasim et al.^28^. The outcome of mixed convection amalgamated with the inclined magnetic field is numerically explored by Mabood et al.^29^ on a second-grade fluid flow past a vertical cylinder with Newtonian heating. Murthy et al.^30^ examined the Casson fluid flow with slip condition and Newtonian heating on a linear stretched cylinder. It is perceived that the temperature of fluid and rate of heat transfer enhances for larger values of Newtonian heating. Suleman et al.^31^ examined the behavior of heat generation/absorption on nanofluid flow over a nonlinear elongated cylinder incorporated with homogeneous and heterogeneous (h–h) reactions. The key outcome of this exploration reveals that the augmentation in the temperature field is noticed by increasing the radiation parameter. Nevertheless, by mounting the (h–h) reaction parameter the concentration field declines.

The variable heat source and sink effects have innumerable applications in the field of engineering and medicine like unpolished oil retrieval, radial diffusers, and cooling of metallic sheets. Rasekh et al.^32^ numerically demonstrated the impact of the variable heat source and sink on a nanofluid flow on a cylindrical surface. It is observed here that by up surging the Brownian and thermophoresis parameters, the surface drag force coefficient declines. Sravanthi^33^ analytically discussed the influence of nonlinear thermal radiation on nanofluid flow on a vertical stretching cylinder with an irregular heat source/sink. Hayat et al.^34^ discussed variable heat source/sink and mixed convection on a Jeffery fluid on an inclined cylinder. By utilizing the analytical approach, it is concluded that the temperature of the fluid is in direct proportionate to the heat source. Lin and Ghaffari^35^ numerically presented the influence of heat transfer on two stretchable disks with variable heat source/sink. Recent analysis involving non-uniform heat source and sink is mentioned in Refs.^36–43^.

The difference in temperature within a system results in the transport of heat from one region to the other. The phenomenon of heat and mass transfer has numerous applications such as heat conduction in tissues, cooling of electronic devices, heat exchangers, food processing, crop damage, power collector, and wire drawing technique. Fourier^44^ formulated a law to understand the transmission of heat in various situations with certain restrictions. The drawback of the Fourier model was that it governs the parabolic equation. Due to which it was insufficient to analyze the behavior of heat flow throughout the medium. Cattaneo^45^ modified Fourier law with the inclusion of relaxation parameter with respect to time. Consequently, this modification results in a hyperbolic energy equation. Christov^46^ added the upper convected Oldroyd derivative to upgrade the Cattaneo model known as Cattaneo–Christov (CC) model. The impact of the CC model on an unsteady Maxwell fluid flow past a stretching cylinder is analyzed by Khan et al.^47^. Shankar and Naduvinamani^48^ numerically examined the characteristics of the CC model on an MHD Casson fluid flow with thermal radiation between two parallel plates. Waqas et al.^49^ addressed the impact of the CC model on a stratified Oldroyd-B fluid flow past an elongated sheet. Khan et al.^50^ focused on Carreau nanofluid flow with the CC model over a paraboloid surface of revolution. Researchers have shown great interest in CC model cited in Refs.^51–53^.

Activation energy is the least energy required by reactants to prompt a chemical reaction. A wide range of utilization of activation energy appears in the preparation of food, hydrodynamics, oil, and water emulsions. In recent years, huge interest is shown by researchers in chemical reactions coupled with heat and mass transfer due to its significance in many processes such as damage of crops due to freezing, drying, food processing, manufacturing of ceramics, and polymer production. An upshot of activation energy on an MHD Casson nanofluid flow over a nonlinear deformed surface is addressed by Shah et al.^19^. It is noticed here that the concentration of nanofluid enhances by escalating the activation energy and reaction rate. Abdelmalek et al.^54^ investigated variable thermal conductivity on a Williamson nanofluid flow with activation energy and second-order slip over a stretching cylinder. It is noticed that the concentration of nanoparticles increases for larger values of activation energy and slip parameters. Activation energy with thermal radiation on an Eyring-Powell nanofluid flow is inspected by Reddy et al.^55^ past an inclined cylinder. It is observed here that drag force decreases for large values of magnetic and curvature parameters. Sarkar et al.^56^ examined the impact of activation energy on a hydromagnetic Sisko nanofluid on a linear stretching cylinder. Lately, researchers have pondered on the fluid flows with activation energy^57–60^.

The above-mentioned literature illustrates that abundant researches are available discussing fluid flow past a linear stretching cylinder. The literature is also available if we talk about the Casson nanofluid flow over the stretched cylinders. But no study so far is attempted that discusses the MHD Casson nanofluid flow with dust particles over a deformable cylinder. The novelty of the envisaged flow model is enhanced with activation energy, binary chemical reaction, and Fourier and Fick’s laws. The flow is analyzed under the impact of variable source/sink and Newtonian heating at the boundary of the cylinder surface. The solution of the formulated mathematical problem is computed by employing bvp4c a built-in function in MATLAB. The aftermath of pertinent parameters is inspected numerically and graphically.

## Mathematical formulation

**Figure 1:**
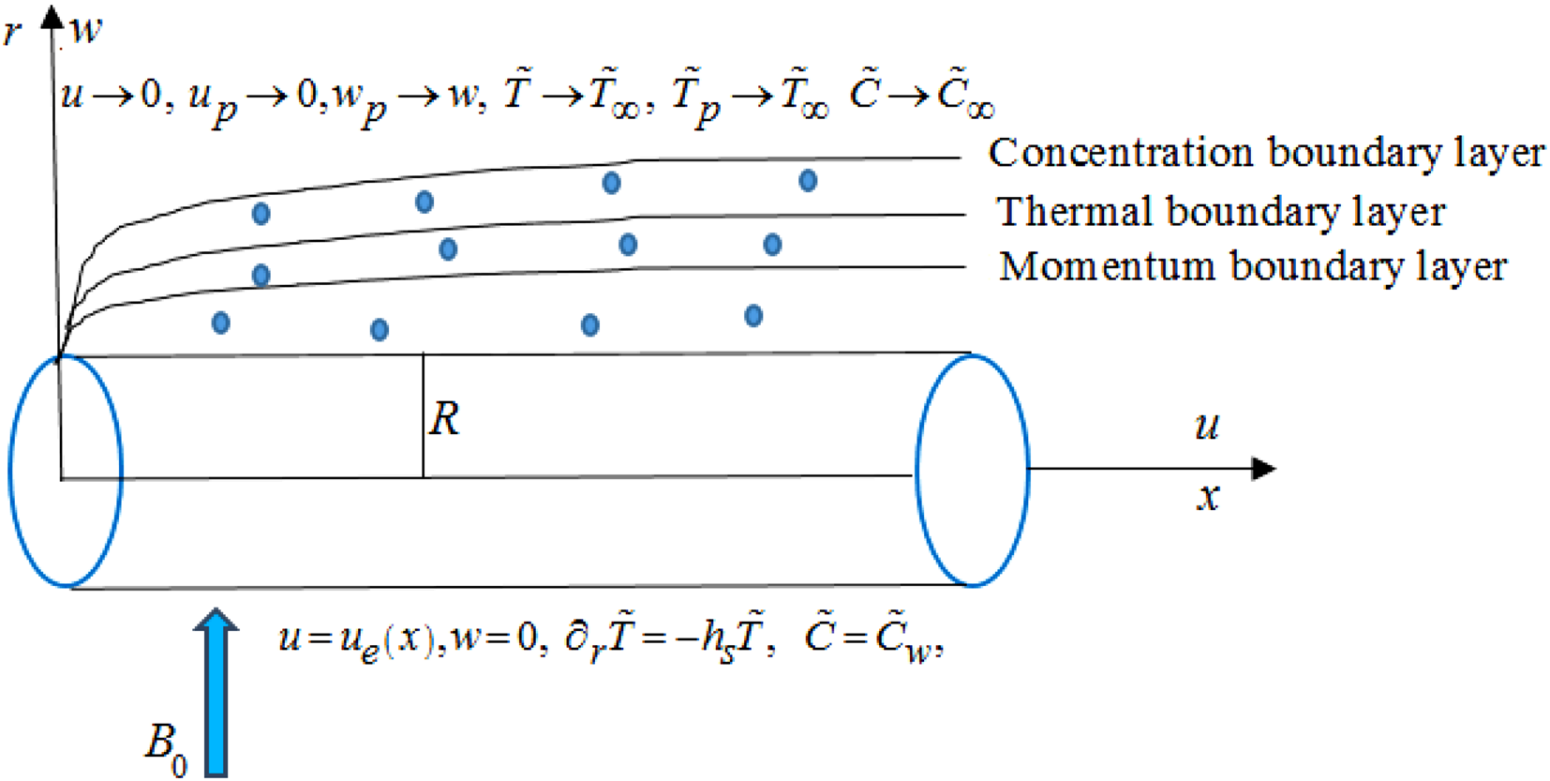
Flow configuration of the model.

An incompressible, two-dimensional MHD dusty Casson nanofluid over a stretching cylinder $r = R$ is considered. Cylindrical coordinates are used. The axis of the cylinder is along the $x$-axis and $r$-axis is perpendicular to the surface of the cylinder. A schematic illustration for the flow is portrayed in Fig. 1. To observe heat and mass diffusion, generalized Fourier, and Fick law is used. The transfer of heat is enhanced by considering the characteristic features of variable heat source/sink and Newtonian heating. The rheological equation for Casson fluid model is demarcated as^61^:

1 $$\tau_{ij} = \left\{ \begin{gathered} \left( {\mu_{c} + \frac{{S_{y} }}{{\left( {2\tilde{\pi }} \right)^{0.5} }}} \right)2\tilde{\gamma }_{ij} , \, if \, \tilde{\pi } > \tilde{\pi }_{c} \hfill \\ \left( {\mu_{c} + \frac{{S_{y} }}{{\left( {2\tilde{\pi }_{c} } \right)^{0.5} }}} \right)2\tilde{\gamma }_{ij} , \, if \, \tilde{\pi } < \tilde{\pi }_{c} \hfill \\ \end{gathered} \right.,$$

where $\tau_{ij}$ is the extra stress tensor, $\tilde{\pi } = \tilde{\gamma }_{ij} \tilde{\gamma }_{ij}$ is the product of the components of deformation rate, $\tilde{\gamma }_{ij} = \frac{1}{2}\left( {\partial_{{x_{j} }} v_{i} + \partial_{{x_{i} }} v_{j} } \right)$ is the rate of the strain tensor, $\tilde{\pi }_{c}$ is the critical value of deformation rate tensor, $S_{y}$ is the fluid yield stress.

The equations associated with the above-stated assumptions are^6,14,30,54^:

For the fluid flow

2 $$\partial_{x} u + \frac{w}{r} + \partial_{r} w = 0,$$

3 $$u\partial_{x} u + w\partial_{r} u = \frac{\nu }{r}\left( {1 + \frac{1}{\beta }} \right)\partial_{r} \left( {r\partial_{r} u} \right) - \frac{{\sigma_{1} B_{0}^{2} }}{\rho }u + \frac{KN}{\rho }\left( {u_{p} - u} \right),$$

4 $$\begin{aligned} & \left( {u\partial_{x} \tilde{T} + w\partial_{r} \tilde{T}} \right) + \varepsilon_{T} \left( \begin{gathered} u^{2} \partial_{xx} \tilde{T} + 2uw\partial_{xr} \tilde{T} + w^{2} \partial_{rr} \tilde{T} + u\partial_{x} u\partial_{x} \tilde{T} \hfill \\ + w\partial_{r} u\partial_{x} \tilde{T} + u\partial_{x} w\partial_{r} \tilde{T} + w\partial_{r} w\partial_{r} \tilde{T} \hfill \\ \end{gathered} \right) = \frac{k}{r}\frac{1}{{\left( {\rho c_{p} } \right)_{f} }}\partial_{r} \left( {r\partial_{r} \tilde{T}} \right) \\ & \quad + \tau \left( {D_{b} \left( {\partial_{r} \tilde{T}\partial_{r} \tilde{C}} \right) + \frac{{D_{t} }}{{\tilde{T}_{\infty } }}\left( {\partial_{r} \tilde{T}} \right)^{2} } \right) + \frac{{\rho_{p} c_{p} }}{{\left( {\rho c_{p} } \right)_{f} \tau_{T} }}\left( {\tilde{T}_{p} - \tilde{T}} \right) + \frac{1}{{\left( {\rho c_{p} } \right)_{f} }}\frac{{ku_{w} }}{x\nu }\left[ {D\left( {\tilde{T}_{\infty } } \right)f^{\prime} + H\left( {\tilde{T} - \tilde{T}_{\infty } } \right)} \right], \\ \end{aligned}$$

5 $$\begin{aligned} & u\partial_{x} \tilde{C} + w\partial_{r} \tilde{C} + \varepsilon_{C} \left( \begin{gathered} u^{2} \partial_{xx} \tilde{C} + 2uw\partial_{xr} \tilde{C} + w^{2} \partial_{rr} \tilde{C} + u\partial_{x} u\partial_{x} \tilde{C} \hfill \\ + w\partial_{r} u\partial_{x} \tilde{C} + u\partial_{x} w\partial_{r} \tilde{C} + w\partial_{r} w\partial_{r} \tilde{C} \hfill \\ \end{gathered} \right) = \frac{{D_{b} }}{r}\partial_{r} \left( {r\partial_{r} \tilde{C}} \right) \\ & \quad + \frac{{D_{t} }}{{\tilde{T}_{\infty } }}\frac{1}{r}\partial_{r} \left( {r\partial_{r} \tilde{T}} \right) - \Lambda^{2} \left( {\tilde{C} - \tilde{C}_{\infty } } \right)\left( {\frac{{\tilde{T}}}{{\tilde{T}_{\infty } }}} \right)^{n} \exp \left( {\frac{{ - E_{a} }}{{k\tilde{T}}}} \right). \\ \end{aligned}$$

For the dusty flow

6 $$\partial_{x} u_{p} + \frac{{w_{p} }}{r} + \partial_{r} w_{p} = 0,$$

7 $$u_{p} \partial_{x} u_{p} + w_{p} \partial_{r} u_{p} = \frac{K}{m}\left( {u - u_{p} } \right),$$

8 $$u_{p} \partial_{x} \tilde{T}_{p} + w_{p} \partial_{r} \tilde{T}_{p} = \frac{{c_{p} }}{{c_{m} \tau_{T} }}\left( {\tilde{T} - \tilde{T}_{p} } \right).$$

with boundary conditions ^3,30,55,62^:

$$\, \left. u \right|_{r = R} = u_{e} \left( x \right) = \frac{{u_{0} x}}{l}, \, \left. w \right|_{r = R} = 0, \, \left. { \, \partial_{r} \tilde{T}} \right|_{r = R} = - h_{s} \tilde{T}, \, \left. {\tilde{C}} \right|_{r = R} = \tilde{C}_{w} ,$$

9 $$\left. u \right|_{r \to \infty } \to {0, }\left. {u_{p} } \right|_{r \to \infty } \to 0,\left. {w_{p} } \right|_{r \to \infty } \to w, \, \left. {\tilde{T}} \right|_{r \to \infty } \to \tilde{T}_{\infty } , \, \left. {\tilde{T}_{p} } \right|_{r \to \infty } \to \tilde{T}_{\infty } \, \left. {\tilde{C}} \right|_{r \to \infty } \to \tilde{C}_{\infty } .$$

Using appropriate transformation^21^:

10 $$\begin{aligned} & u = \frac{{u_{0} x}}{l}f^{\prime}\left( \zeta \right), \, w = - \left( {\frac{{u_{0} v}}{l}} \right)^{1/2} .\frac{R}{r}f\left( \zeta \right), \, \zeta = \left( {\frac{{u_{0} }}{\nu l}} \right)^{1/2} \left( {\frac{{r^{2} - R^{2} }}{2R}} \right), \, u_{p} = \frac{{u_{0} x}}{l}F^{\prime}\left( \zeta \right), \\ & w_{p} = - \left( {\frac{{u_{0} v}}{l}} \right)^{1/2} .\frac{R}{r}F\left( \zeta \right), \, \tilde{T} = \tilde{T}_{\infty } \, \theta \left( \zeta \right) + \tilde{T}_{\infty } , \, \tilde{T}_{p} = \tilde{T}_{\infty } \, \theta_{p} \left( \zeta \right) + \tilde{T}_{\infty } , \, \tilde{C} = \left( {\tilde{C}_{w} - \tilde{C}_{\infty } } \right) \, \phi \left( \zeta \right) + \tilde{C}_{\infty } . \\ \end{aligned}$$

By utilizing the above transformation, the continuity Eqs. (2) and (6) are satisfied. However, Eqs. (3)–(5) and (7)–(8) are transmuted into dimensionless form:

For the fluid flow

11 $$\begin{aligned} \left( {1 + {\raise0.7ex\hbox{$1$} \!\mathord{\left/ {\vphantom {1 \beta }}\right.\kern-\nulldelimiterspace} \!\lower0.7ex\hbox{$\beta $}}} \right) \, \left[ {\left( {1 + 2\omega \zeta } \right) \, \frac{{d^{3} f}}{{d\zeta^{3} }} + 2\omega \, \frac{{d^{2} f}}{{d\zeta^{2} }}} \right] & = - Ha \, \left( {\frac{df}{{d\zeta }}} \right) + \lambda \delta_{v} \, \left( {\frac{dF}{{d\zeta }} - \frac{df}{{d\zeta }}} \right) \\ & \quad - \left( {\frac{df}{{d\zeta }}} \right)^{2} + f \, \frac{{d^{2} f}}{{d\zeta^{2} }} = 0, \\ \end{aligned}$$

12 $$\begin{aligned} & \left( {1 + 2\omega \zeta } \right) \, \frac{{d^{2} \theta }}{{d\zeta^{2} }} + 2\omega \, \frac{d\theta }{{d\zeta }} + D\frac{df}{{d\zeta }} + H\theta + \Pr \, \left( {1 + 2\omega \zeta } \right)\left( {N_{t} \, \left( {\frac{d\theta }{{d\zeta }}} \right)^{2} + N_{b} \, \frac{d\theta }{{d\zeta }} \, \frac{d\phi }{{d\zeta }}} \right) \\ & \quad + \Pr \left( {f\frac{d\theta }{{d\zeta }} - K_{1} \left( {f^{2} \frac{{d^{2} \theta }}{{d\zeta^{2} }} + f\frac{df}{{d\zeta }}\frac{d\theta }{{d\zeta }}} \right) + \lambda \delta_{T} \left( {\theta_{p} - \theta } \right)} \right) = 0, \\ \end{aligned}$$

13 $$\begin{aligned} & \left( {1 + 2\omega \zeta } \right) \, \frac{{d^{2} \phi }}{{d\zeta^{2} }} + 2\omega \, \frac{d\phi }{{d\zeta }} - S_{c} K_{2} \left( {f^{2} \, \frac{{d^{2} \phi }}{{d\zeta^{2} }} + f \, \frac{df}{{d\zeta }} \, \frac{d\phi }{{d\zeta }}} \right) \\ & \quad - \delta \phi S_{c} \left( {1 + \theta } \right)^{n} \exp \left( {\frac{ - E}{{1 + \theta }}} \right) + S_{c} f \, \frac{d\phi }{{d\zeta }} + \frac{{N_{t} }}{{N_{b} }} \, \left( {2\omega \, \frac{d\theta }{{d\zeta }} + \left( {1 + 2\omega \zeta } \right) \, \frac{{d^{2} \theta }}{{d\zeta^{2} }}} \right) = 0. \\ \end{aligned}$$

For the dusty flow

14 $$F\frac{{d^{2} F}}{{d\zeta^{2} }} + \delta_{v} \left( {\frac{df}{{d\zeta }} - \frac{dF}{{d\zeta }}} \right) - \left( {\frac{dF}{{d\zeta }}} \right)^{2} = 0,$$

15 $$F\frac{{d\theta_{p} }}{d\zeta } + \gamma \delta_{T} \left( {\theta - \theta_{p} } \right) = 0.$$

and the modified boundary conditions are:

16 $$\begin{aligned} & f(\zeta ) = 0, \, \frac{df}{{d\zeta }} = 1, \, \frac{d\theta }{{d\zeta }}\left( \zeta \right) = - A \, \left( {1 + \theta \left( \zeta \right)} \right), \, \phi (\zeta ) = 1\,{\text{at}}\,\zeta = 0 \, \\ & \frac{df}{{d\zeta }} \to 0, \, \frac{dF}{{d\zeta }} \to 0, \, F(\zeta ) + f(\zeta ) = 0, \, \theta \left( \zeta \right) \to 0, \, \theta_{p} \left( \zeta \right) \to 0, \, \phi (\zeta ) \to 0 \, \,{\text{as}}\,\zeta \to \infty . \\ \end{aligned}$$

The drag force coefficient $C_{f}$ temperature gradient $Nu_{x}$ and rate of mass transfer $Sh_{x}$ on the wall are specified as:

17 $$C_{f} = \frac{{2\tau_{w} }}{{\rho u_{w}^{2} }}\quad \tau_{w} = \mu \left( {1 + \frac{1}{\beta }} \right)\left( {\partial_{r} u} \right)_{r = R} ,$$

18 $$Nu_{x} = \frac{{xQ_{w} }}{{k\left( {\tilde{T} - \tilde{T}_{\infty } } \right)}} \, \quad Q_{w} = - k\left( {\partial_{r} \tilde{T}} \right)_{r = R} ,$$

19 $$Sh_{x} = \frac{{xQ_{m} }}{{D_{b} \left( {\tilde{C}_{w} - \tilde{C}_{\infty } } \right)}} \, \quad Q_{m} = - D_{b} \left( {\partial_{r} \tilde{C}} \right)_{r = R} .$$

By utilizing Eqs. (10), (17)–(19) are transmuted as:

20 $$\frac{1}{2} \, C_{f} {\text{Re}}_{x}^{0.5} = \left( {1 + \frac{1}{\beta }} \right)\left. { \, \frac{{d^{2} f}}{{d\zeta^{2} }}} \right|_{\zeta = 0} ,$$

21 $$\frac{{Nu_{x} }}{{\left( {{\text{Re}}_{x} } \right)^{1/2} }} = A \, \left( {1 + \frac{1}{\theta (0)}} \right),$$

22 $$\frac{{Sh_{x} }}{{\left( {{\text{Re}}_{x} } \right)^{1/2} }} = - \left. {\frac{d\phi }{{d\zeta }}} \right|_{\zeta = 0} .$$

## Numerical solution

The exact solution of the ODEs (11)–(15), with the boundary conditions (16) is not possible as these are highly nonlinear coupled equations. It is solved numerically using MATLAB software bvp4c technique.

23 $$\begin{aligned} & f = Y_{1} ,f^{\prime} = Y_{2} ,f^{\prime\prime} = Y_{3} ,f^{\prime\prime\prime} = Y_{3}^{\prime } = YY_{1} ,F = Y_{4} ,F^{\prime} = Y_{5} ,F^{\prime\prime} = Y_{5}^{\prime } = YY_{2} , \\ & YY_{1} = \frac{1}{{\left( {1 + 2\omega \zeta } \right)\left( {1 + {\raise0.7ex\hbox{$1$} \!\mathord{\left/ {\vphantom {1 \beta }}\right.\kern-\nulldelimiterspace} \!\lower0.7ex\hbox{$\beta $}}} \right)}}\left[ {Y_{2}^{2} - Y_{1} .Y_{3} + Ha.Y_{2} - \lambda \delta_{v} .\left( {Y_{5} - Y_{2} } \right)} \right] - \frac{2\omega }{{\left( {1 + 2\omega \zeta } \right)}}.Y_{3} , \\ & YY_{2} = \frac{{\left[ {Y_{5}^{2} - \delta_{v} .\left( {Y_{2} - Y_{5} } \right)} \right]}}{{Y_{4} }}, \\ & \theta = Y_{6} ,\theta^{\prime} = Y_{7} ,\theta^{\prime\prime} = Y_{7}^{\prime } = YY_{3} ,\theta_{p} = Y_{8} ,\theta_{p}^{\prime } = Y_{8}^{\prime } = YY_{4} , \\ & YY_{3} = \frac{1}{{\left( {1 + 2\omega \zeta } \right) - \Pr .K_{1} .Y_{1}^{2} }}\left[ \begin{gathered} - 2\omega .Y_{7} - \Pr \left( \begin{gathered} \left( {1 + 2\omega \zeta } \right)\left( {N_{b} .Y_{7} .Y_{10} + N_{t} .Y_{7}^{2} } \right) \hfill \\ - Y_{1} .Y_{7} + K_{1} .Y_{1} .Y_{2} .Y_{7} - \lambda \delta_{T} \left( {Y_{8} - Y_{6} } \right) \hfill \\ \end{gathered} \right) \hfill \\ - D.Y_{2} - H.Y_{6} \hfill \\ \end{gathered} \right], \\ & YY_{4} = \frac{{\left[ { - \gamma \delta_{T} .\left( {Y_{6} - Y_{8} } \right)} \right]}}{{Y_{4} }}, \\ & \phi = Y_{9} ,\phi^{\prime} = Y_{10} ,\phi^{\prime\prime} = Y_{10}^{\prime } = YY_{5} , \\ & YY_{5} = \frac{1}{{\left( {1 + 2\omega \zeta } \right) - S_{c} .K_{2} .Y_{1}^{2} }}\left[ \begin{gathered} - 2\omega .Y_{10} - S_{c} \left( \begin{gathered} Y_{1} .Y_{10} - K_{2} .Y_{1} .Y_{2} .Y_{10} \hfill \\ - \delta .Y_{9} \left( {1 + \alpha .Y_{6} } \right)^{n} \exp \left( {\frac{ - E}{{\left( {1 + \alpha .Y_{6} } \right)}}} \right) \hfill \\ \end{gathered} \right) \hfill \\ - \frac{{N_{t} }}{{N_{b} }}\left( {\left( {1 + 2\omega \zeta } \right).YY_{3} + 2\omega .Y_{7} } \right) \hfill \\ \end{gathered} \right] \\ & {\text{and}}\,{\text{the}}\,{\text{boundary}}\,{\text{conditions}}\,(16)\,{\text{are}}\,{\text{enumerated}}\,{\text{as}} \\ & Y_{1} (0) = 0,Y_{2} (0) = 1,Y_{7} (0) = - A(1 + Y_{6} (0)),Y_{9} (0) = 1\,{\text{at}}\,\zeta = 0 \\ & Y_{2} (\infty ) \to 0,Y_{5} (\infty ) \to 0,Y_{4} (\infty ) + Y_{1} (\infty ) \to 0,Y_{6} (\infty ) \to 0,Y_{8} (\infty ) \to 0,Y_{9} (\infty ) \to 0\,{\text{as}}\,\zeta \to \infty . \\ \end{aligned}$$

Table 1 shows the comparison of $Nu_{x} {\text{Re}}_{x}^{ - 0.5}$ with Upadhya^6^ and Murthy^30^ for varied estimates of Pr by fixing $K_{1} = N_{t} = N_{b} = D = H = S_{c} = K = E = n = 0$. An excellent agreement between the values is attained.

**Table 1:** Comparison of $Nu_{x} {\text{Re}}_{x}^{ - 0.5}$ for current analysis with Upadhya^6^ and Murthy^30^.

Pr	$Nu_{x} {\text{Re}}_{x}^{ - 0.5}$		
	Upadhya^6^	Murthy^30^	Present
0.72	1.08862	1.088642	1.088632
1	1.33333	1.333333	1.333333
10	4.79584	4.796929	4.796346

## Graphical results and discussion

**Figure 2:**
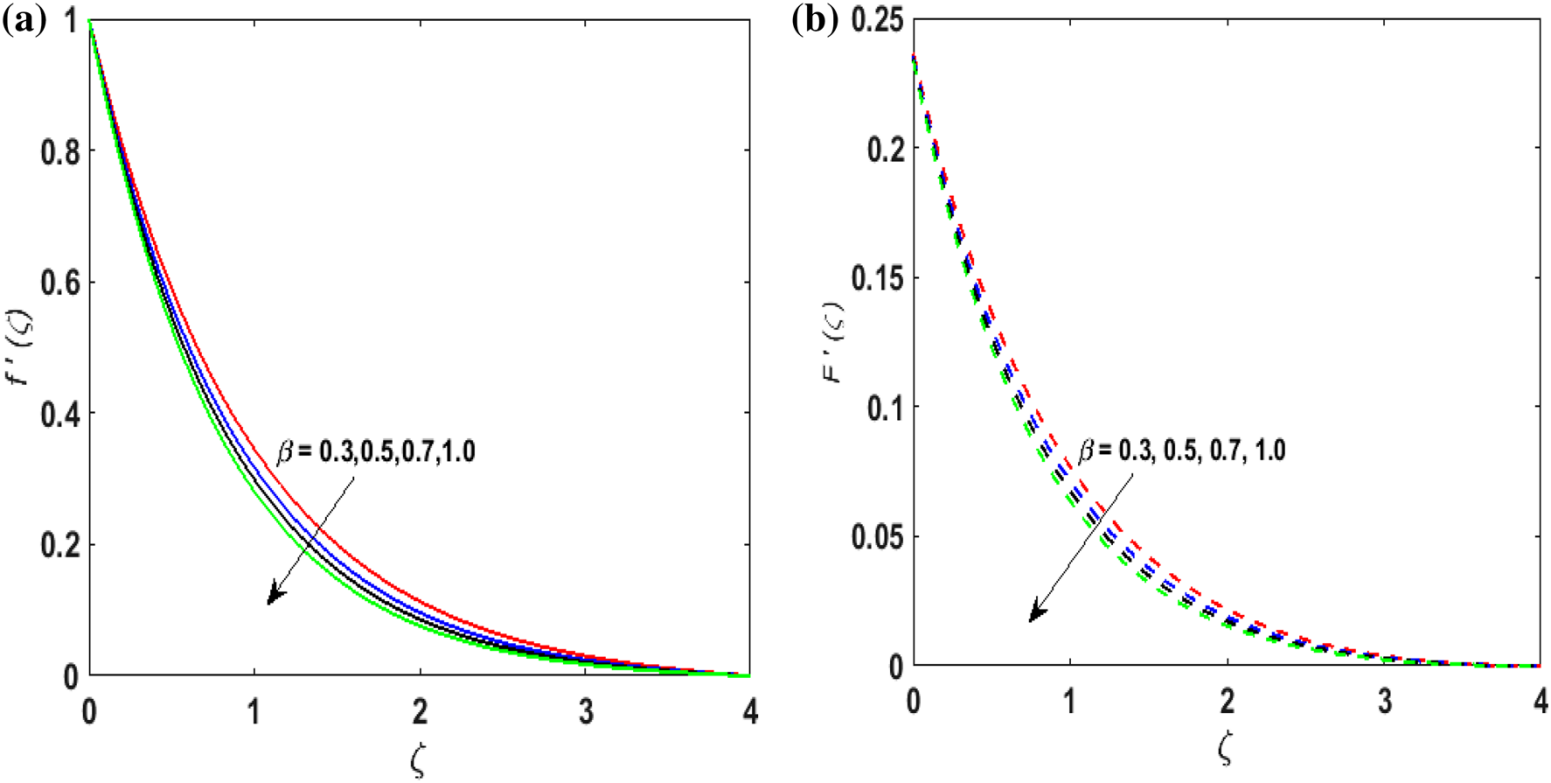
(**a**) $f^{\prime}\left( \zeta \right)$ for various $\beta$. (**b**) $F^{\prime}\left( \zeta \right)$ for various $\beta$.

**Figure 3:**
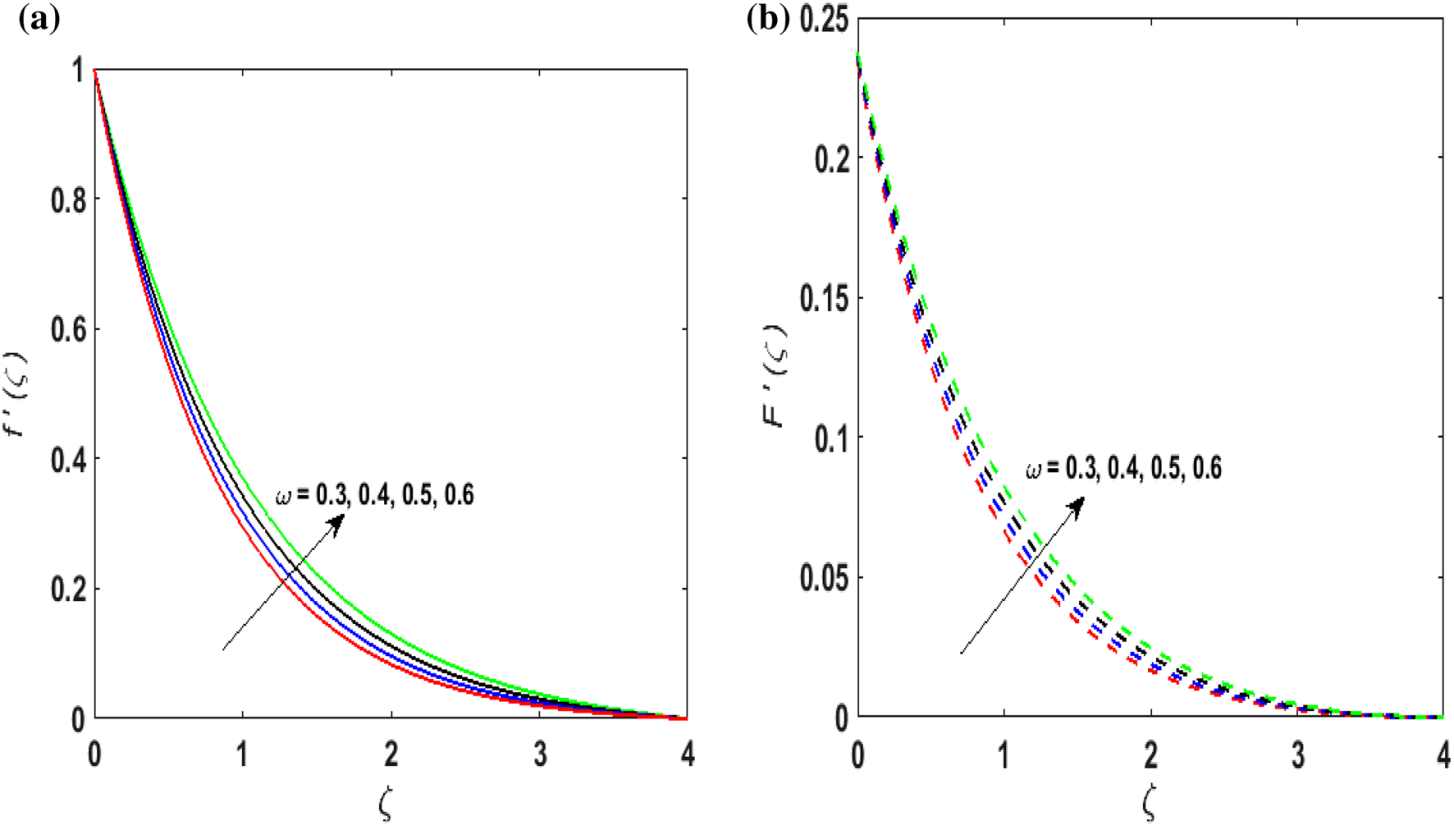
(**a**) $f^{\prime}\left( \zeta \right)$ for various $\omega .$ (**b**) $F^{\prime}\left( \zeta \right)$ for various $\omega .$

**Figure 4:**
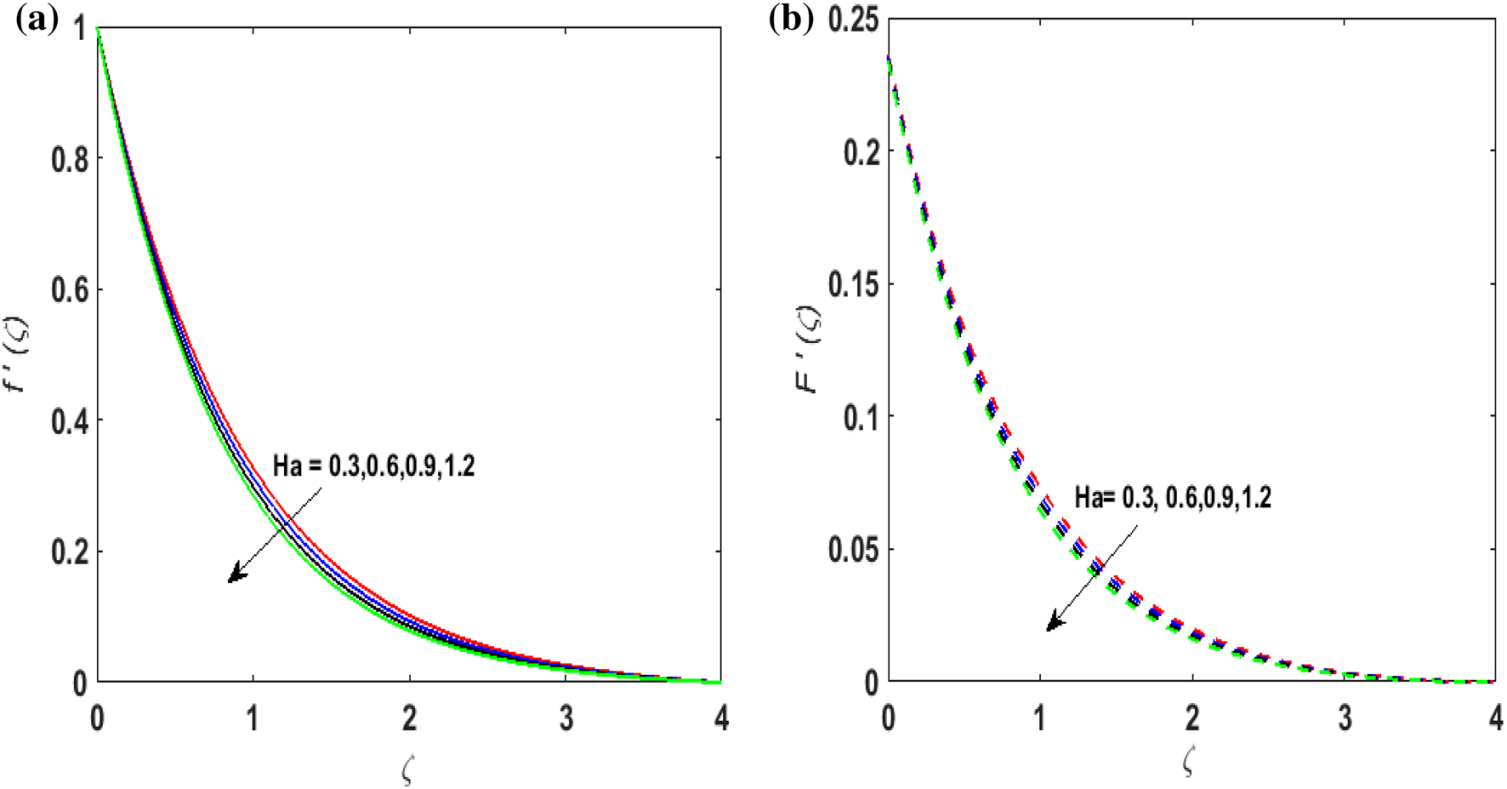
(**a**) $f^{\prime}\left( \zeta \right)$ for various $Ha.$ (**b**) $F^{\prime}\left( \zeta \right)$ for various $Ha.$

**Figure 5:**
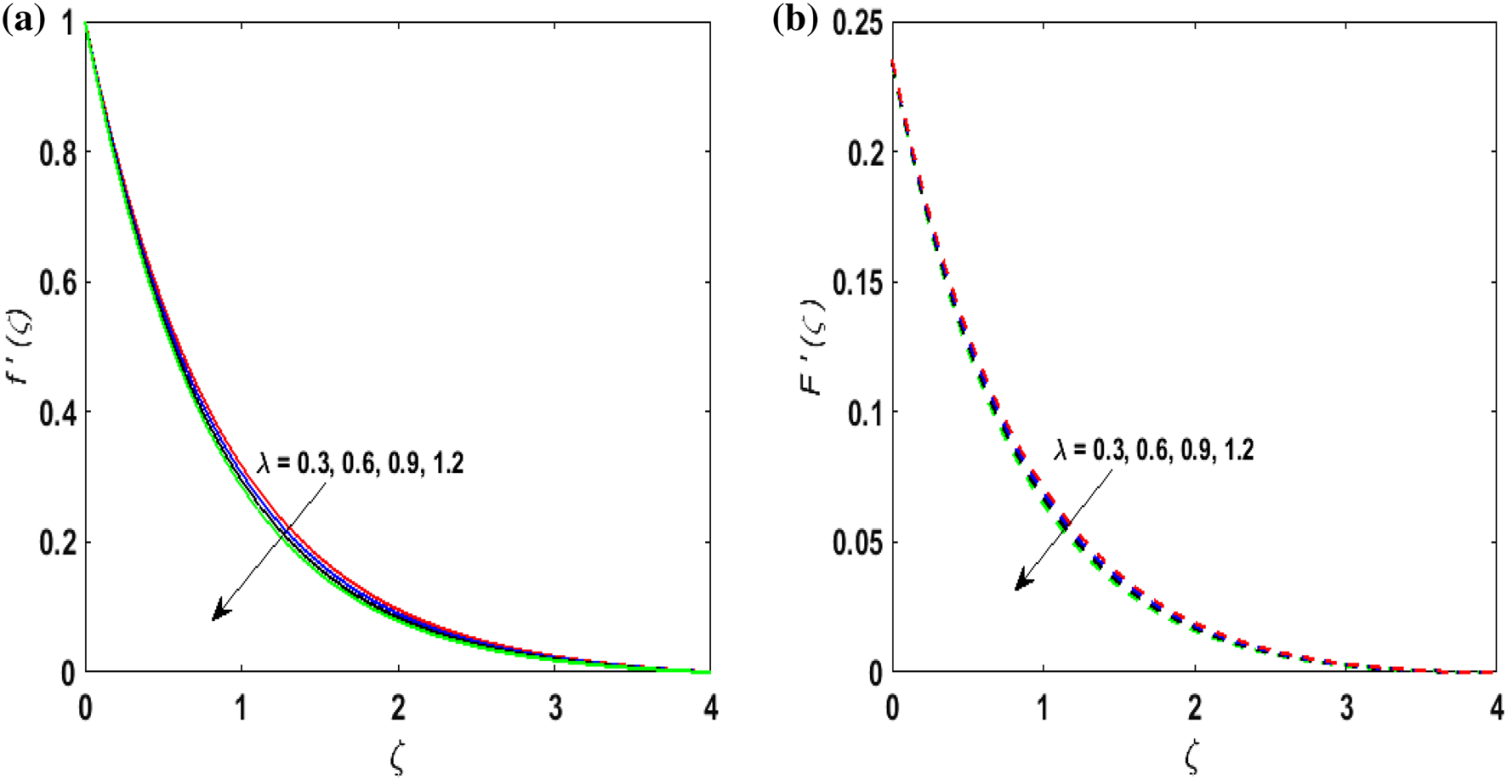
(**a**) $f^{\prime}\left( \zeta \right)$ for various $\lambda .$ (**b**) $F^{\prime}\left( \zeta \right)$ for various $\lambda .$

**Figure 6:**
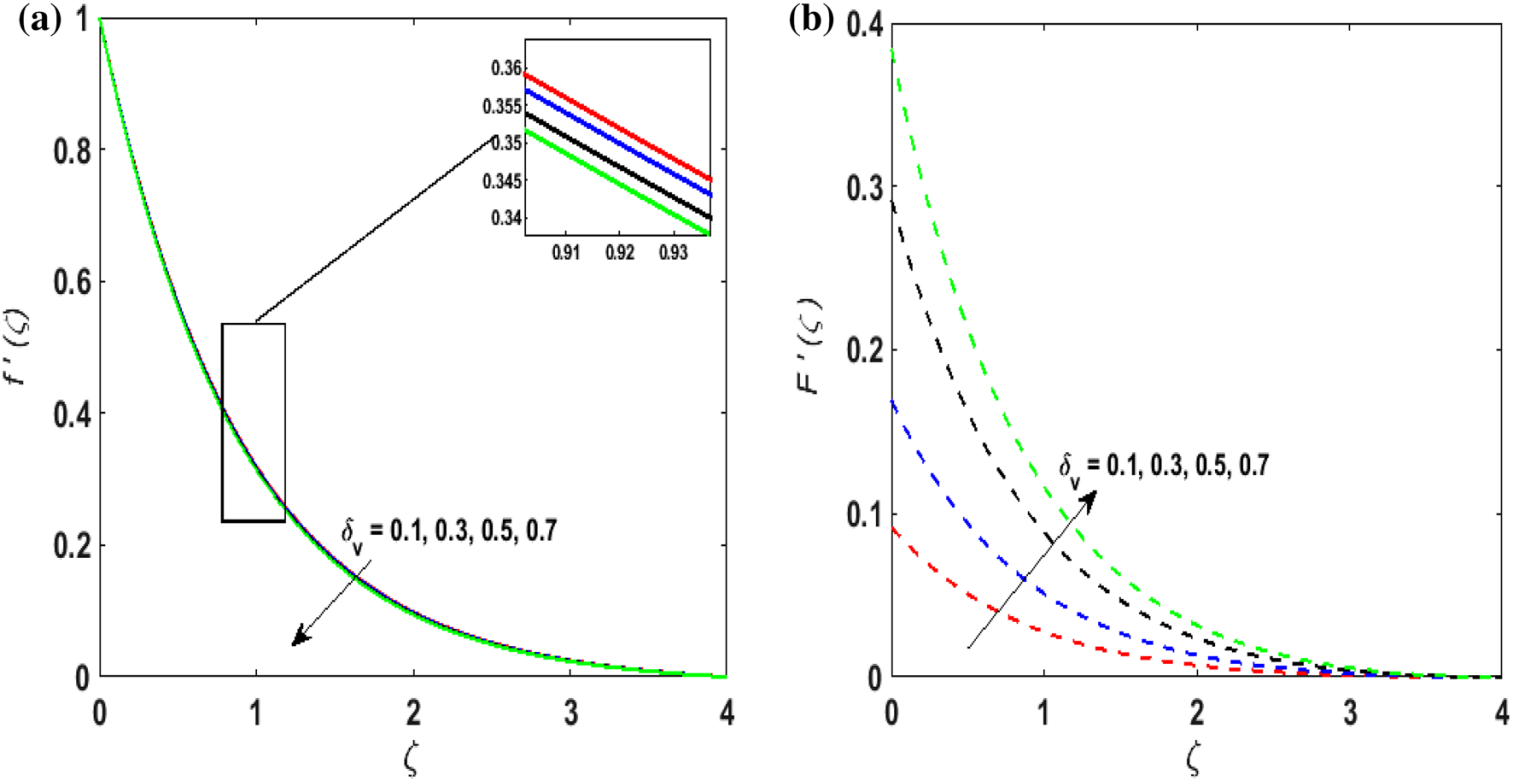
(**a**) $f^{\prime}\left( \zeta \right)$ for various $\delta_{v} .$ (**b**) $F^{\prime}\left( \zeta \right)$ for various $\delta_{v} .$

For the graphical results of highly nonlinear mathematical problem in Eqs. (11)–(15) bvp4c and implemented function in MATLAB with the imposed boundary conditions (16) are utilized. Our foremost emphasis is to analyze the behavior of fluid-particle suspension for various parameters on the fluid flow and temperature field. The impact of chemical reaction with activation energy and Fick law on the concentration field is discussed. Numeric values of dimensionless parameters are taken as $$0.3 \le \beta \le 0.7, \, 0.2 \le \omega \le 0.6,$$$$0.1 \le Ha \le 0.7, \, 0.2 \le \lambda \le 0.6,$$$$0.2 \le \delta_{v} \le 0.7,$$$${2} \le \Pr \le 10, \, 0.2 \le K_{1} \le 1, \, 0.1 \le N_{b} \le 0.8,$$$$0.1 \le N_{t} \le 0.6,$$$$0.1 \le D \le 0.5,$$$$0.4 \le \gamma \le 0.8,$$$$0.5 \le S_{c} \le 1.2, \, 0 \le H \le 0.5, \, 0.4 \le K_{2} \le 1, \, 0.6 \le E \le 1,$$$$0 \le A \le 0.4{\text{ and }}0.2 \le \delta \le 0.6.$$ Figure 2a,b exhibits the behavior of Casson fluid parameter $$\beta$$ on the velocity field $$f^{\prime}\left( \zeta \right)$$ and suspended particle phase $$F^{\prime}\left( \zeta \right)$$. As $$\beta$$ is in direct proportionate to the dynamic viscosity and inverse proportionate to the yield stress $$S_{y}$$ of Casson fluid. By increasing $$\beta$$ the yield stress $$S_{y}$$ decreases. For growing values of $$\beta$$, viscosity generates frictional force. This opposes the fluid flow. It is observed that due to escalation in $$\beta ,$$ momentum boundary layer thickness degenerates and a deteriorating nature is observed by the velocity field of both phases. Figure 3a,b are sketched to analyze the effect of curvature parameter $$\omega$$ on $$f^{\prime}\left( \zeta \right)$$ and $$F^{\prime}\left( \zeta \right)$$. As the radius of the cylinder $$R$$ is inverse proportionate to the curvature parameter $$\omega$$. By upsurging $$\omega$$, a diminution is noticed in the radius of the cylinder. The contact of the surface area of the cylinder with the fluid decreases. Hence, the velocity profile is enhanced as less resistance is offered to the flow of fluid. Figure 4a,b explains the influence of the magnetic parameter $$Ha$$ on the velocity field $$f^{\prime}\left( \zeta \right)$$ and $$F^{\prime}\left( \zeta \right).$$ On enlarging $$Ha$$, Lorentz force is produced. As higher values of $$Ha$$ strengthens the Lorentz force. This force opposes the motion of the fluid. This force tends to reduce fluid velocity. Consequently, a downfall is noticed in the velocity of both the dusty and fluid phases. Figure 5a,b illustrates the behavior of $$\lambda$$ on the velocity field $$f^{\prime}\left( \zeta \right)$$ and $$F^{\prime}\left( \zeta \right)$$. It is perceived that by increasing $$\lambda$$, the drag force increases which results in hindrance to the movement of the fluid. Thus, velocities $$f^{\prime}\left( \zeta \right)$$ and $$F^{\prime}\left( \zeta \right)$$ declines. Figure 6a,b show how the fluid-particle interaction parameter affects the velocity profiles $$f^{\prime}\left( \zeta \right)$$ and $$F^{\prime}\left( \zeta \right).$$ It is perceived that on augmenting $$\delta_{v}$$ velocity field $$f^{\prime}\left( \zeta \right)$$ diminishes, whereas, a reverse outcome is noticed for $$F^{\prime}\left( \zeta \right)$$. This is because interaction amid the suspended particles and fluid is high. Thus, suspended particles develop a force that opposes the fluid phase unless the velocity of the dusty particles is close to the fluid velocity. Therefore, on escalating the fluid-particle interaction parameter velocity of the suspended particles uprises, however, fluid velocity depreciates.

**Figure 7:**
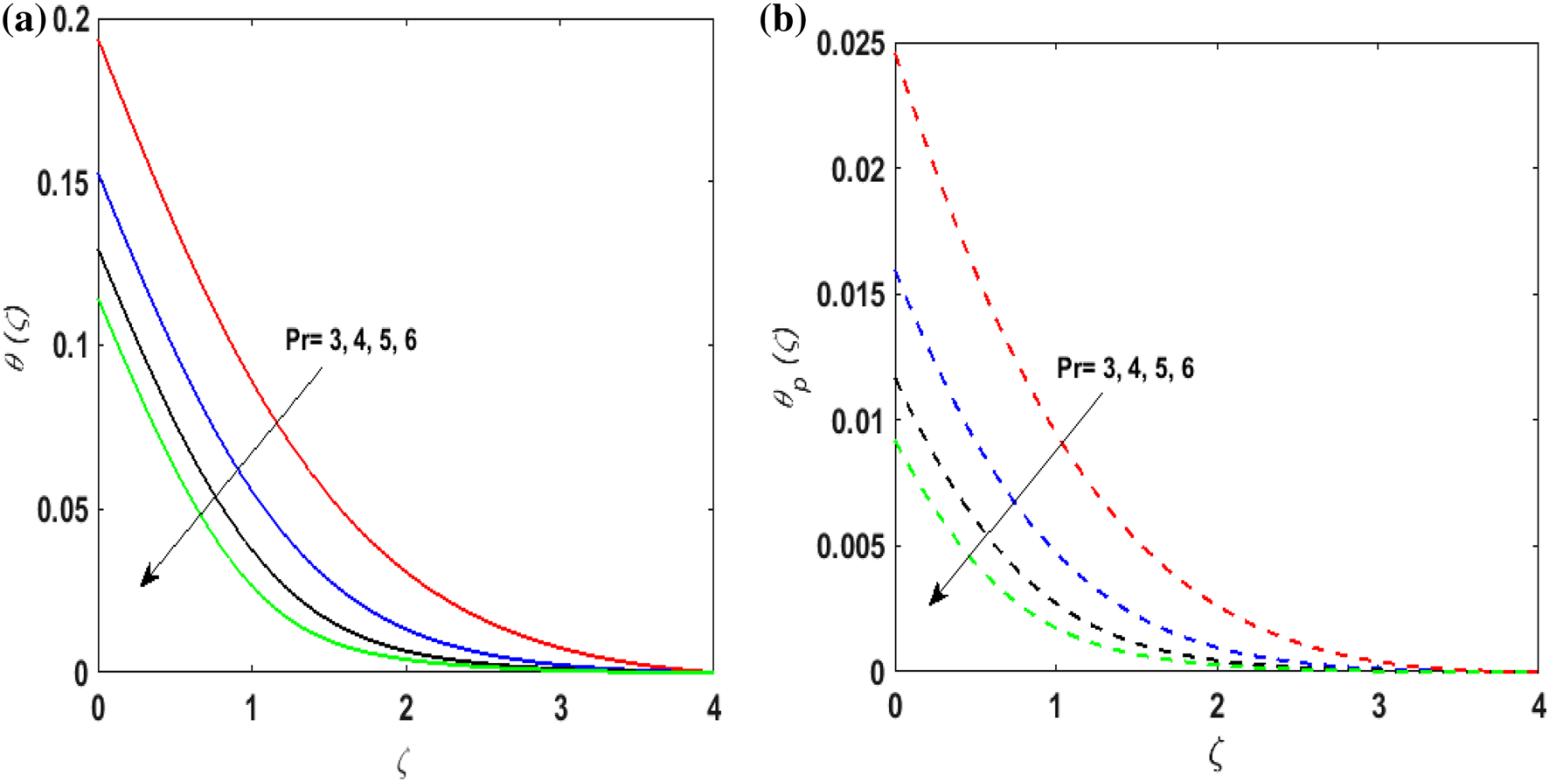
(**a**) $\theta \left( \zeta \right)$ for various $\Pr .$ (**b**) $\theta_{p} \left( \zeta \right)$ for various $\Pr .$

**Figure 8:**
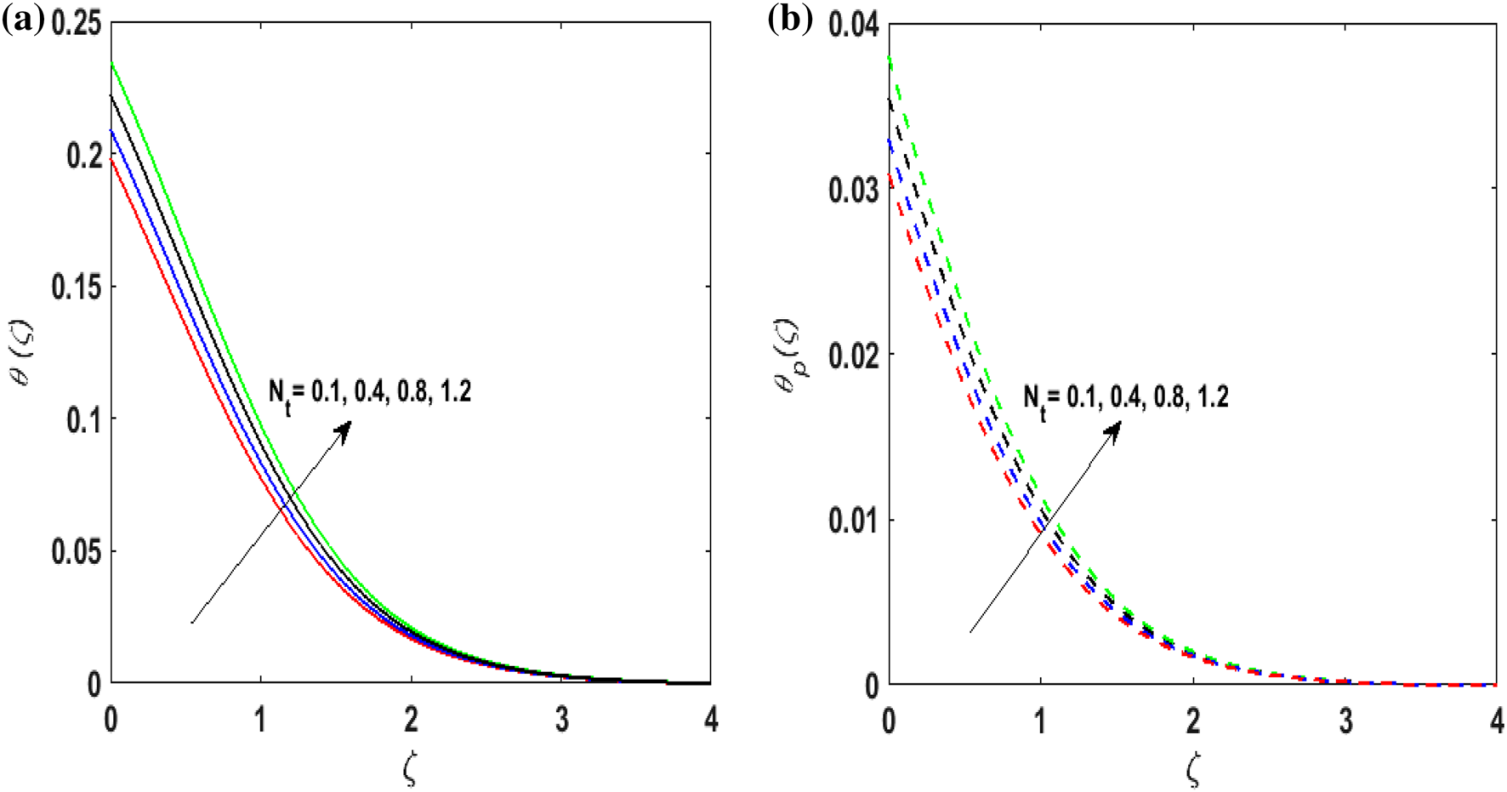
(**a**) $\theta \left( \zeta \right)$ for various $N_{t} .$ (**b**) $\theta_{p} \left( \zeta \right)$ for various $N_{t} .$

**Figure 9:**
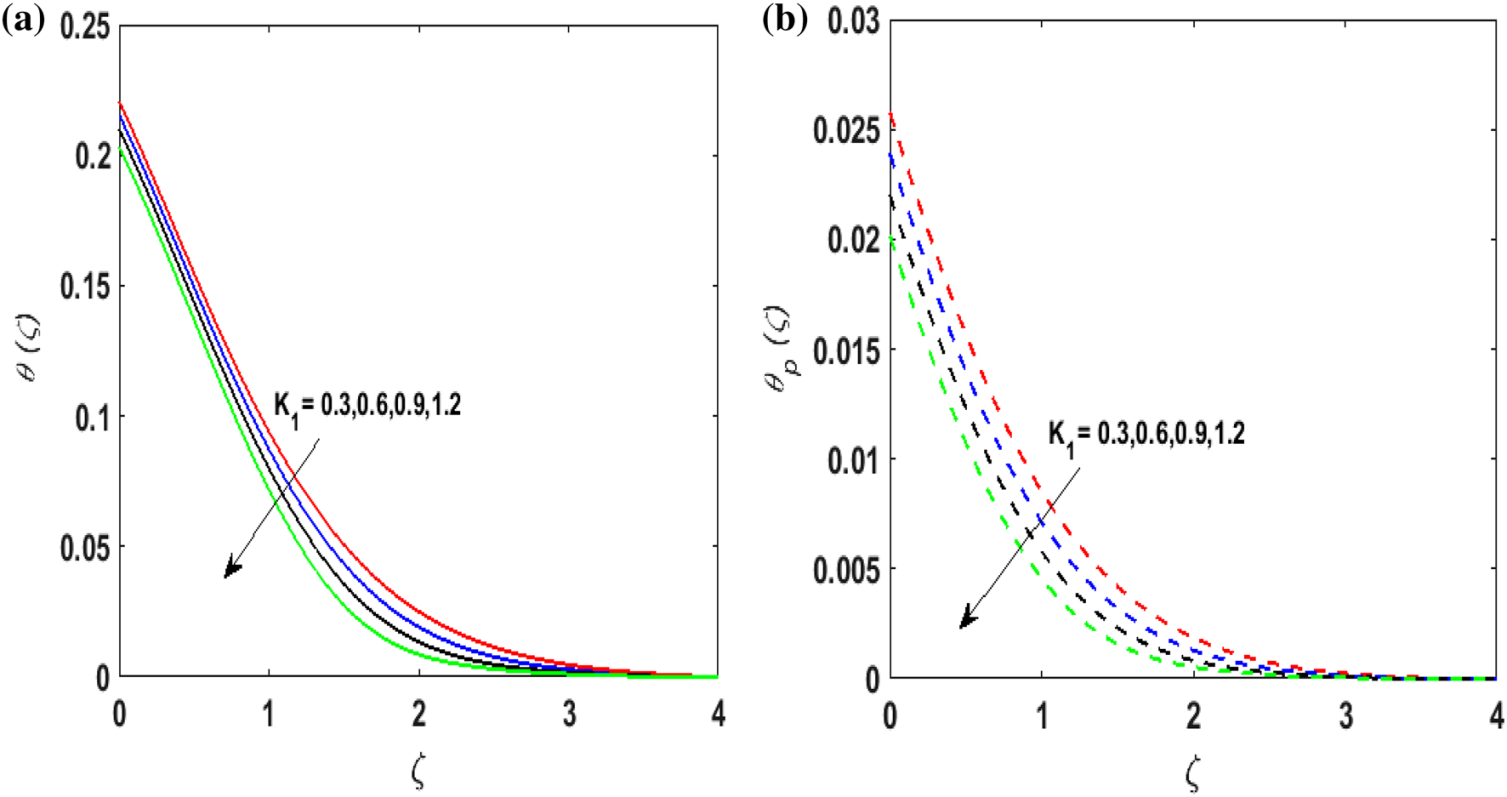
(**a**) $\theta \left( \zeta \right)$ for various $K_{1} .$ (**b**) $\theta_{p} \left( \zeta \right)$ for various $K_{1} .$

**Figure 10:**
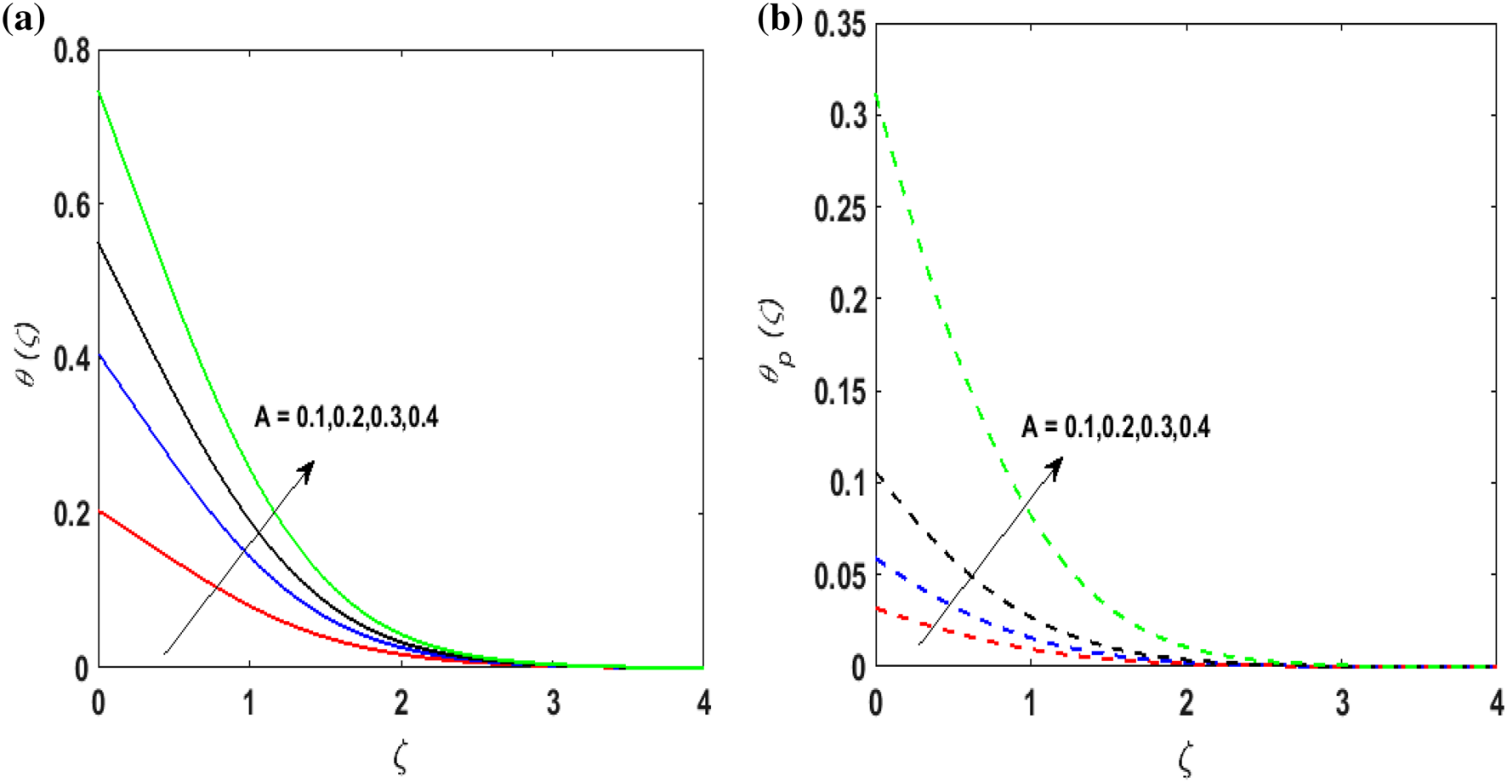
(**a**) $\theta \left( \zeta \right)$ for various $A.$ (**b**) $\theta_{p} \left( \zeta \right)$ for various $A.$

**Figure 11:**
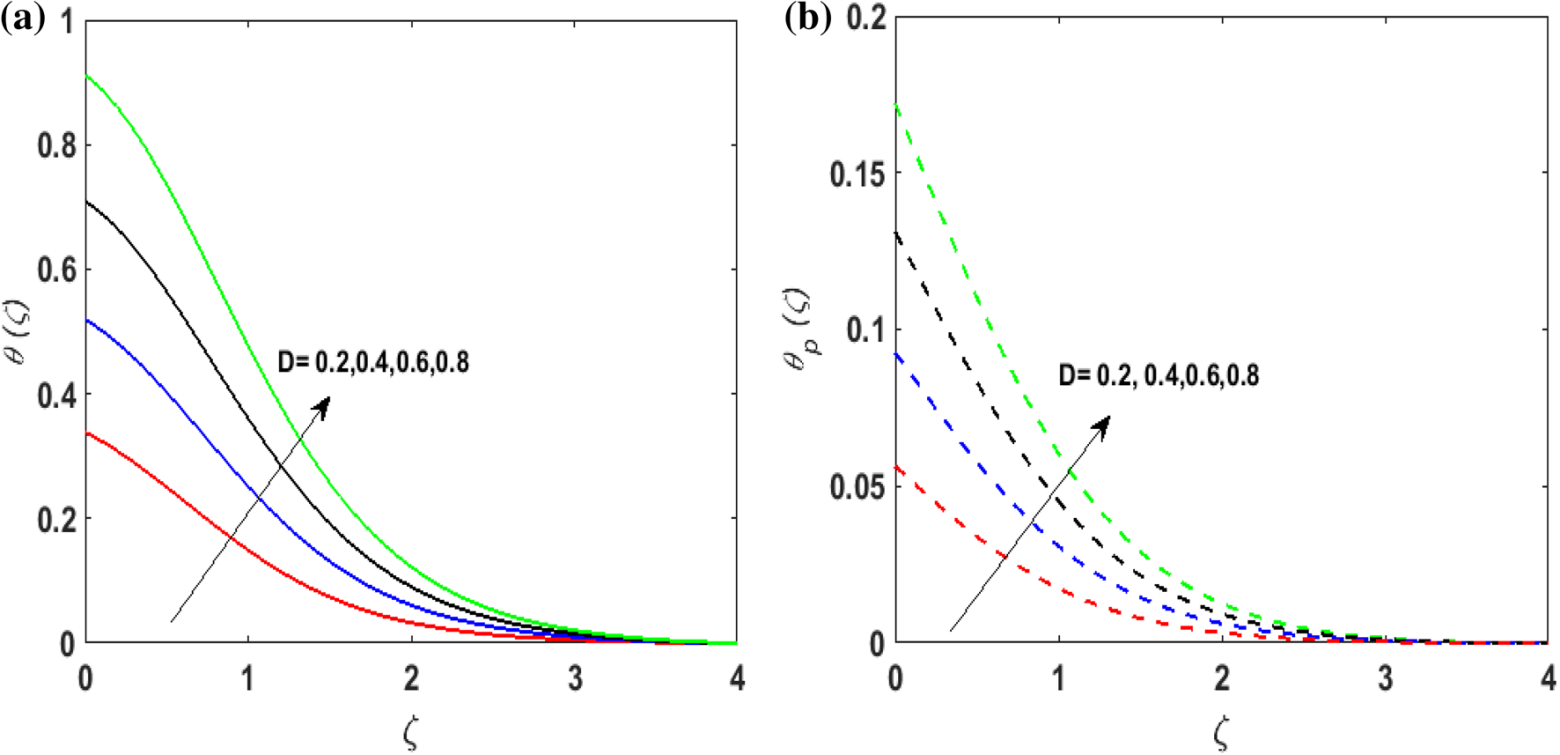
(**a**) $\theta \left( \zeta \right)$ for various $D > 0.$ (**b**) $\theta_{p} \left( \zeta \right)$ for various $D > 0.$

**Figure 12:**
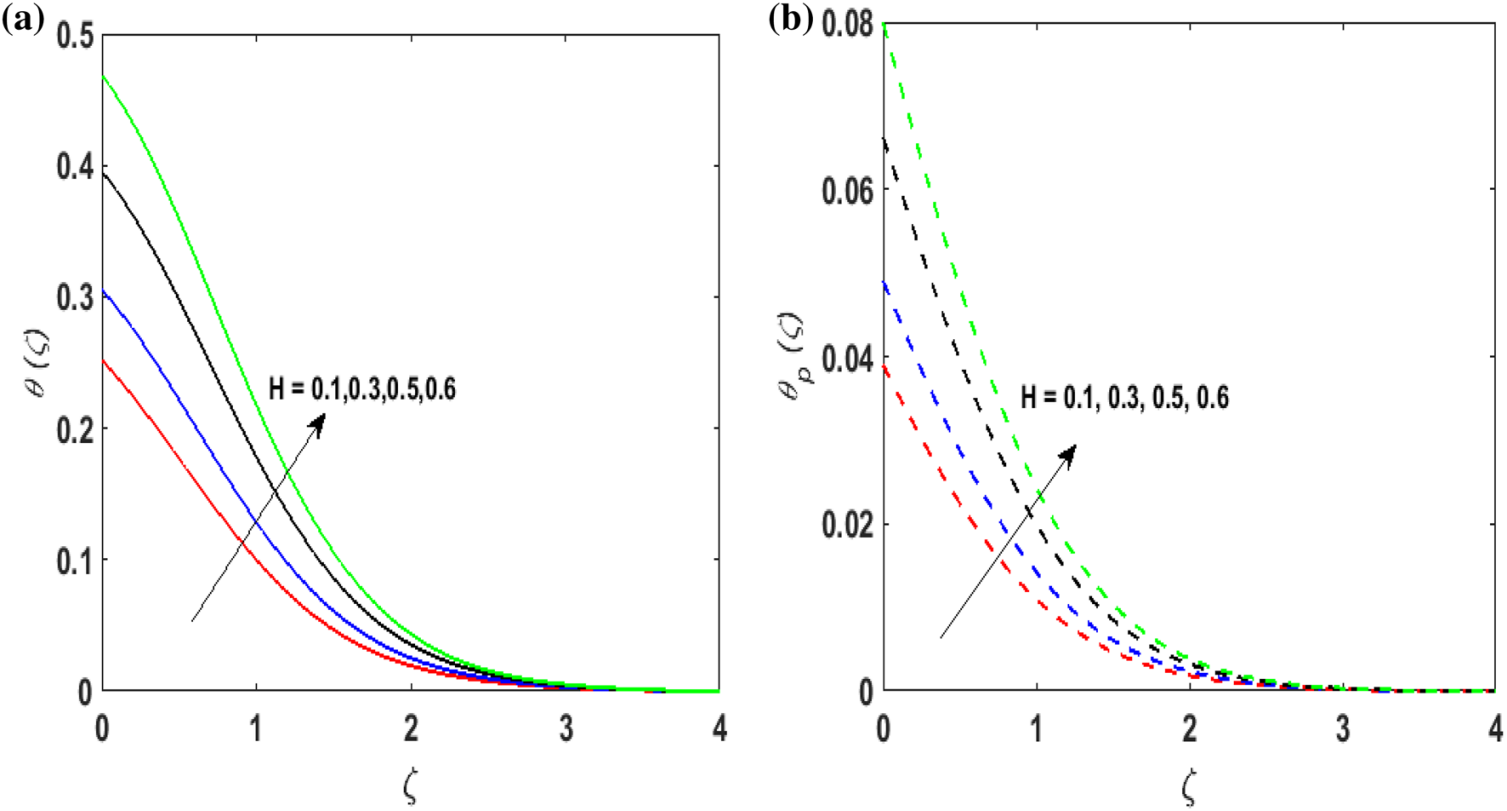
(**a**) $\theta \left( \zeta \right)$ for various $H > 0.$ (**b**) $\theta_{p} \left( \zeta \right)$ for various $H > 0.$

**Figure 13:**
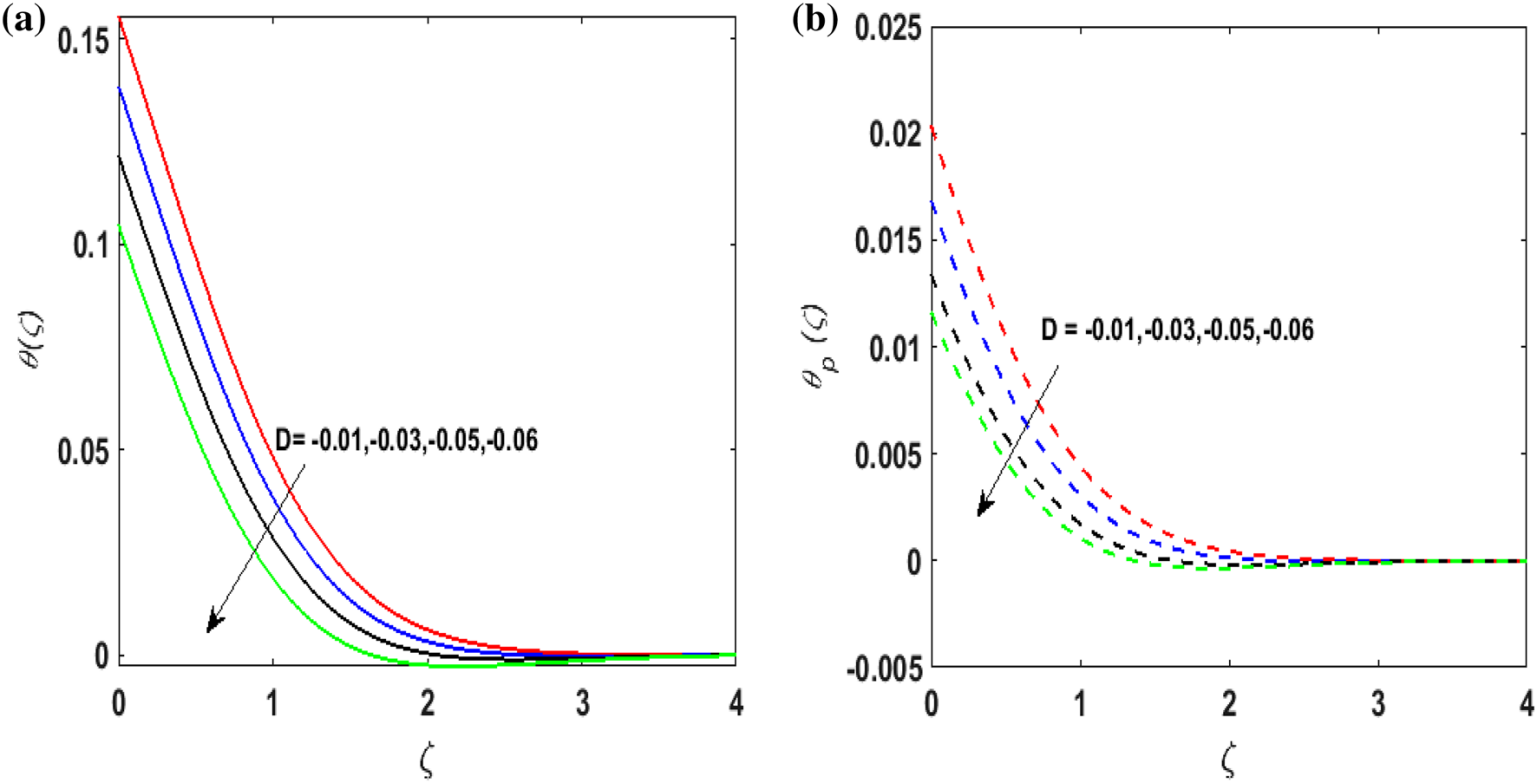
(**a**) $\theta \left( \zeta \right)$ for various $D < 0.$ (**b**) $\theta_{p} \left( \zeta \right)$ for various $D < 0.$

**Figure 14:**
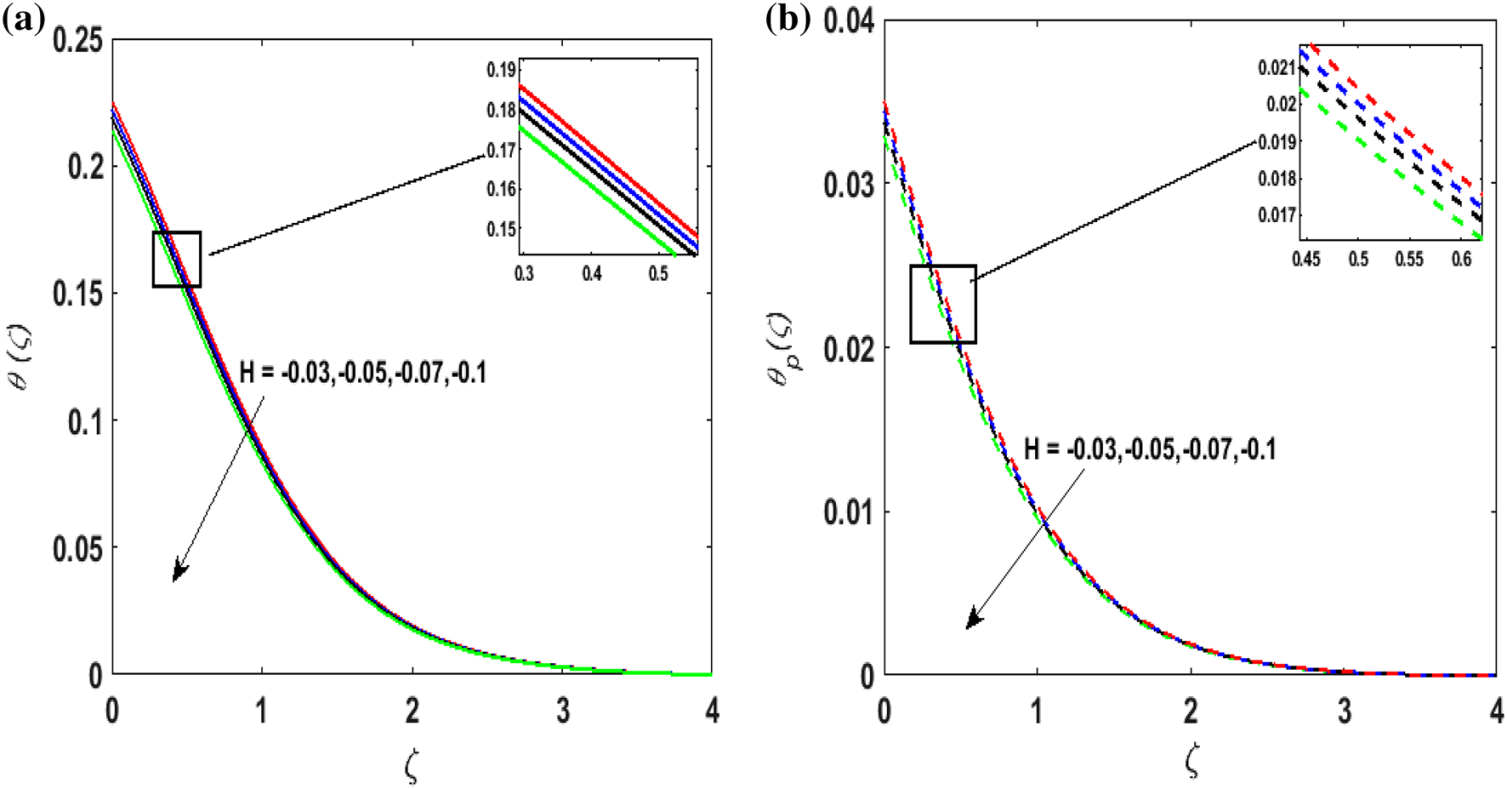
(**a**) $\theta \left( \zeta \right)$ for various $H < 0.$ (**b**) $\theta_{p} \left( \zeta \right)$ for various $H < 0.$

Figure 7a,b portrays the characteristics of the Prandtl number $\Pr$ on the temperature profile $\theta \left( \zeta \right){\text{ and }}\theta_{p} \left( \zeta \right)$ for both phases. Since $\Pr = \frac{{\mu c_{p} }}{k}$ so by varying $\Pr$ thermal diffusion declines. This results in the thinning of the thermal boundary layer and $\theta \left( \zeta \right){\text{ and }}\theta_{p} \left( \zeta \right)$ decreases. In Fig. 8a,b, the outcome of the features of $N_{t}$ on $\theta \left( \zeta \right){\text{ and }}\theta_{p} \left( \zeta \right)$ is depicted. On amplifying $N_{t}$, the temperature of the fluid far away from the surface upsurges. Therefore, $\theta \left( \zeta \right){\text{ and }}\theta_{p} \left( \zeta \right)$ augments. Figure 9a,b portrays the outcome of thermal relaxation time $K_{1}$ on $\theta \left( \zeta \right){\text{ and }}\theta_{p} \left( \zeta \right)$. As the relaxation parameter is enhanced an additional time is required it for the transmission of energy from the heated surface to the fluid. Thus, the thermal relaxation parameter assesses the time for the transmission of heat. Therefore, escalating values of $K_{1}$ deteriorates $\theta \left( \zeta \right){\text{ and }}\theta_{p} \left( \zeta \right)$. To visualize the impact of the conjugate heat parameter $A$ on $\theta \left( \zeta \right){\text{ and }}\theta_{p} \left( \zeta \right)$ Fig. 10a,b is plotted. Higher values of $A$ boosts the rate of heat transfer. This is because more heat is transferred from the hot surface of the cylinder to the cold fluid. Subsequently, fluid temperature increases and this elevates $\theta \left( \zeta \right){\text{ and }}\theta_{p} \left( \zeta \right)$ and thermal boundary layer thickness. The influence of the variable source parameter on $\theta \left( \zeta \right){\text{ and }}\theta_{p} \left( \zeta \right)$ is discussed in Figs. 11a,b and 12a,b. For larger values of $D > 0,H > 0$ more heat is produced as they correspond to the internal heat source. This uplifts the thermal boundary layer as it generates energy for positive values of $D > 0,H > 0$ . Consequently, $\theta \left( \zeta \right){\text{ and }}\theta_{p} \left( \zeta \right)$ increases. Figures 13a,b and 14a,b portrays the influence of variable heat sink on the thermal field $\theta \left( \zeta \right){\text{ and }}\theta_{p} \left( \zeta \right)$. As $D < 0,H < 0$ behave as an internal heat absorber which controls the transfer of heat in the fluid flow. Thus, the thermal boundary layer declines. Hence a deteriorating nature is exhibited by $\theta \left( \zeta \right){\text{ and }}\theta_{p} \left( \zeta \right)$.

**Figure 15:**
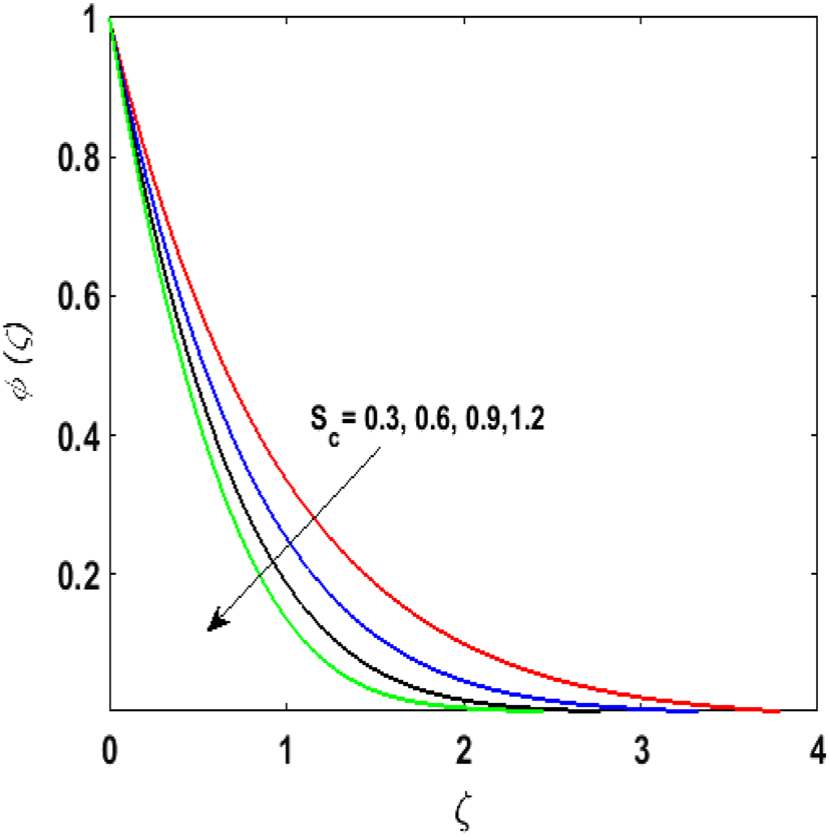
$$\phi \left( \zeta \right){\text{ for various}}$$$$S_{c} .$$

**Figure 16:**
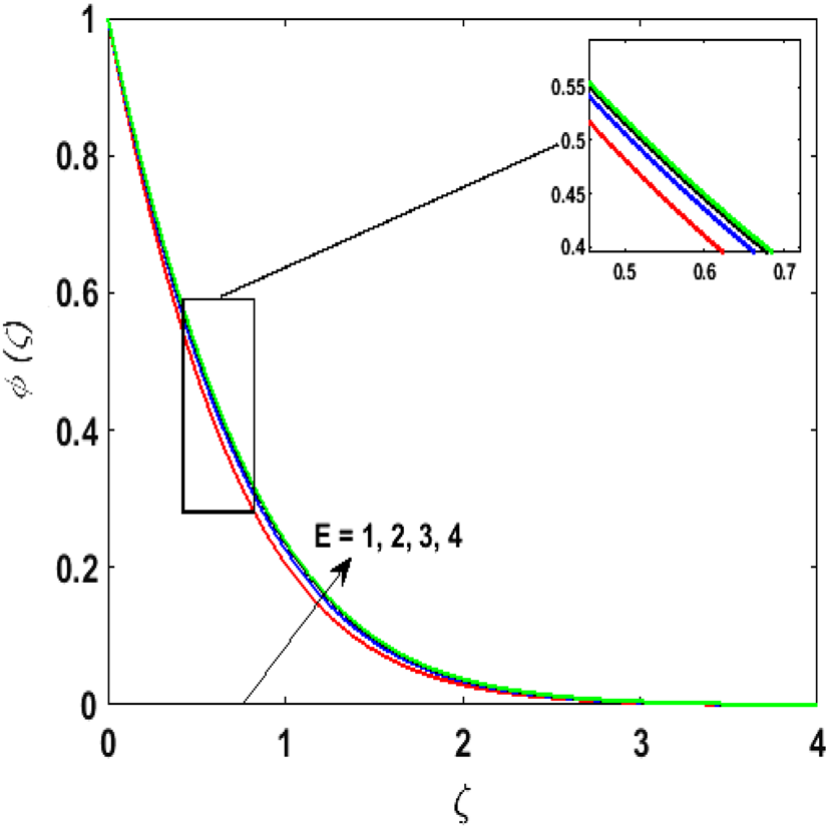
$$\phi \left( \zeta \right){\text{ for various}}$$$$E.$$

**Figure 17:**
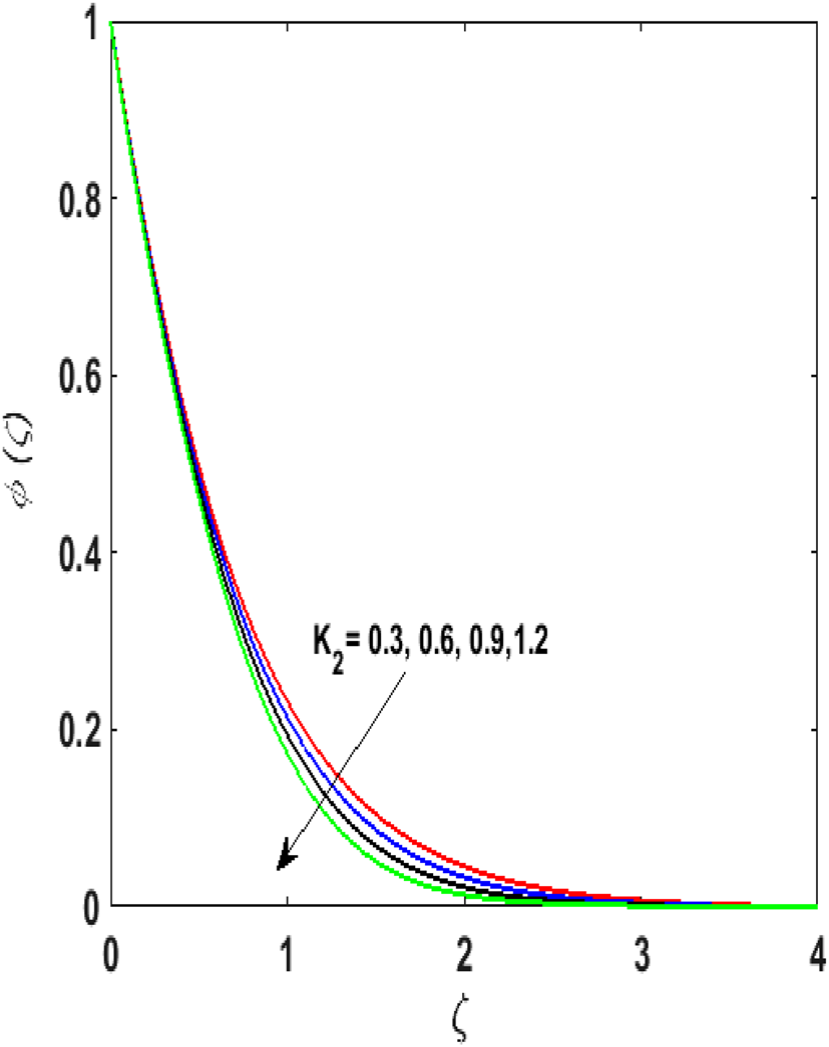
$$\phi \left( \zeta \right){\text{ for various}}$$$$K_{2} .$$

**Figure 18:**
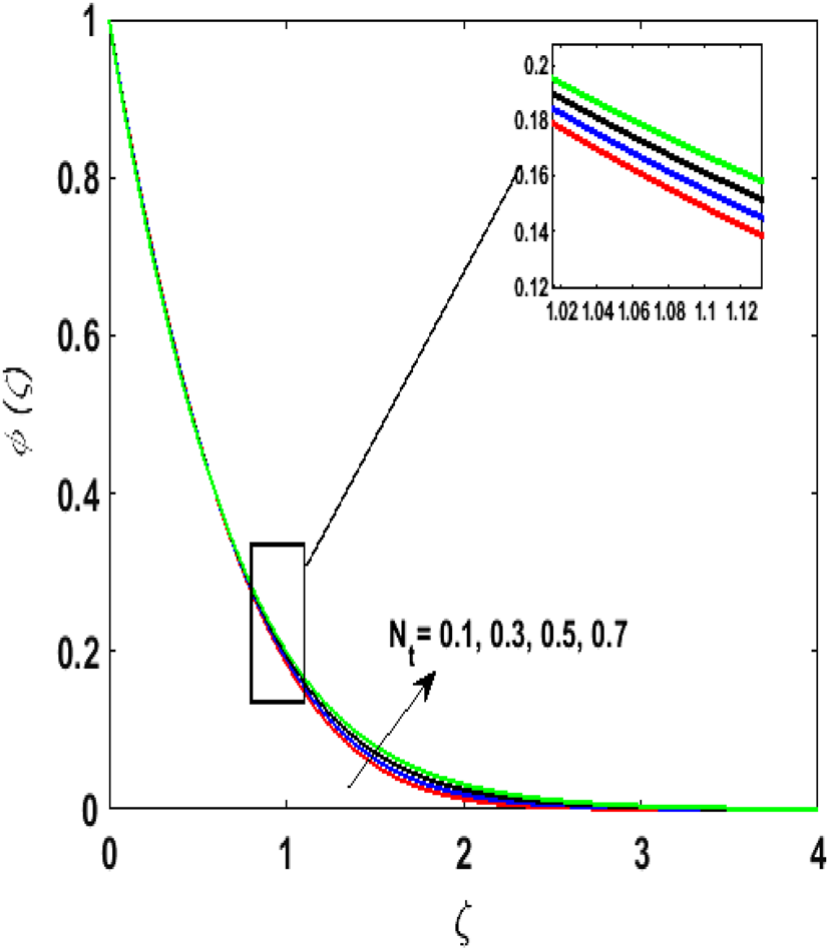
$$\phi \left( \zeta \right){\text{ for various}}$$$$N_{t} .$$

**Figure 19:**
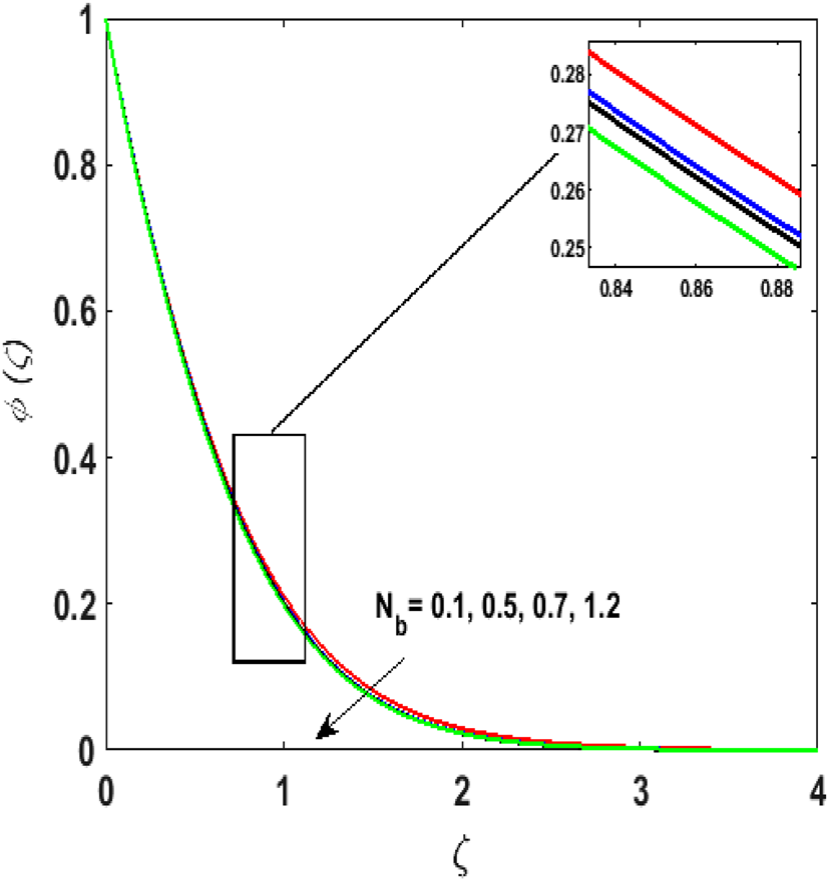
$$\phi \left( \zeta \right){\text{ for various}}$$$$N_{b} .$$

**Figure 20:**
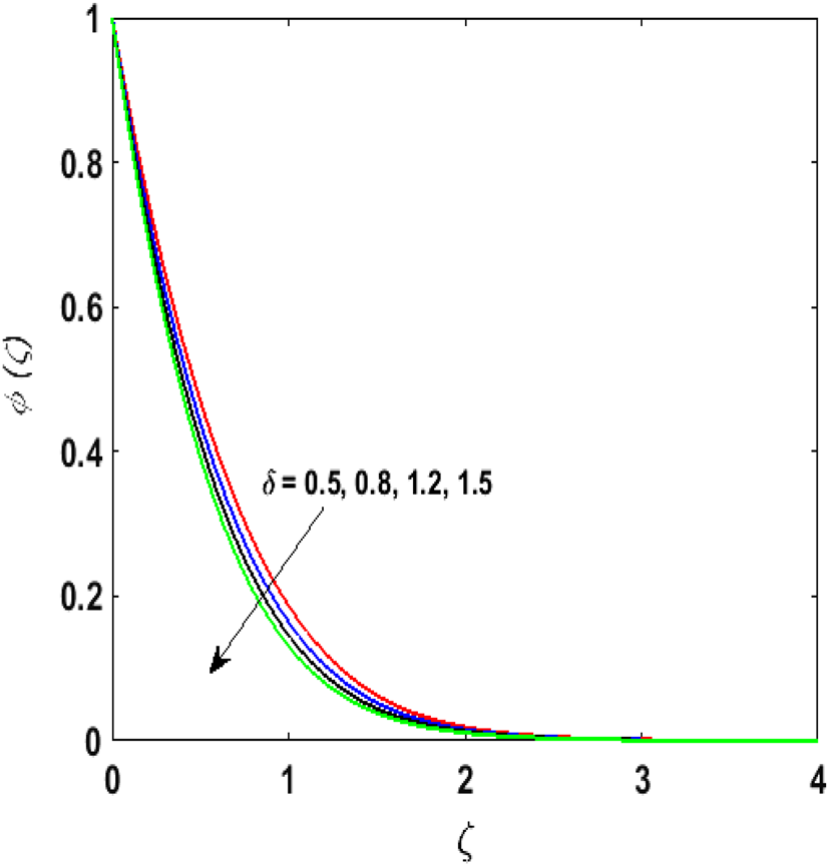
$$\phi \left( \zeta \right){\text{ for various}}$$$$\delta .$$

Figure 15 examines the aftermath of the Schmidt number $$S_{c}$$ on the concentration profile $$\phi (\zeta )$$. Schmidt number is the ratio of viscosity to mass diffusivity. On boosting $$S_{c}$$, a reduction in mass diffusion is noticed. It is perceived that fluids with amplified $$S_{c}$$ corresponds to small mass diffusion. Thus, $$\phi (\zeta )$$ decays. Figure 16 depicts the aftermath of $$E$$ on $$\phi (\zeta )$$. The fluid concentration is enhanced for large estimates of the $$E.$$ Large values of $$E$$ results in a decrease in the value of the expression $$e^{{\left( {{\raise0.7ex\hbox{${ - E}$} \!\mathord{\left/ {\vphantom {{ - E} {1 + \alpha \theta }}}\right.\kern-\nulldelimiterspace} \!\lower0.7ex\hbox{${1 + \alpha \theta }$}}} \right)}}$$. This leads to a minimum reaction rate and therefore slows down the chemical reaction. Thus, increasing $$\phi (\zeta ).$$ To understand the influence of concentration relaxation time $$K_{2}$$ on $$\phi (\zeta )$$ Fig. 17 is sketched. By increasing $$K_{2}$$, more time is entailed by fluid particles to diffuse through the material medium. Hence, $$\phi (\zeta )$$ decreases. Figures 18 and 19 are sketched to witness the impact of the Brownian motion $$N_{b}$$ and thermophoresis parameter $$N_{t}$$ on $$\phi (\zeta )$$. An opposing trend is noticed for $$N_{b}$$ and $$N_{t}$$ versus $$\phi (\zeta ).$$ Large values of $$N_{t}$$ strengthens the movement of particles and it enhances the $$\phi (\zeta )$$. By increasing $$N_{b}$$, within the boundary fluid becomes warm and exacerbates the random motion of particles. Therefore, higher values of $$N_{b}$$ abates the $$\phi (\zeta )$$. Figure 20 portrays the impact of the chemical reaction parameter $$\delta$$ on $$\phi (\zeta )$$. Growing values of $$\delta$$ result in a reduction in chemical molecular diffusivity. By increasing $$\delta$$ a slight decrement is noticed in the boundary layer thickness. Hence, $$\phi (\zeta )$$ represses.

An outstanding correlation of the present result is found for numeric values of temperature gradient in Table 1 with Upadhya et al.^6^ and Murthy et al.^30^. Table 2 portrays the drag force coefficient numerically for distinct values of $\beta ,\delta_{v} {\text{ and }}Ha$. Growing values of $\delta_{v} {\text{ and }}Ha$ augments skin friction, whereas, an opposite impact is observed for $\beta$. Table 3 displays the behavior of $A,\Pr ,K_{1} ,N_{b} ,N_{t}$ on temperature gradient at the surface. By mounting $A,\Pr {\text{ and }}K_{1}$, the rate of heat transfer escalates. However, $Nu{\text{Re}}_{x}^{ - 0.5}$ deteriorates for higher values of $N_{b} {\text{ and }}N_{t} .$ Table 4 depicts the outcome of numerous values of $N_{b} ,N_{t} ,K_{2} {\text{ and }}S_{c}$ on $Sh{\text{Re}}_{x}^{ - 0.5}$. It is noted that $Sh_{x} {\text{Re}}_{x}^{ - 0.5}$ amplifies for larger values of $N_{b} ,N_{t} ,K_{2} {\text{ and }}S_{c}$.

**Table 2:** Numeric values of $$\left( {1 + \frac{1}{\beta }} \right)\left. { \, \frac{{d^{2} f}}{{d\zeta^{2} }}} \right|_{\zeta = 0}$$ for distinct values of $$\beta ,Ha,\delta_{v}$$ when $$\omega = \lambda = 0.5,\Pr = 2,K_{1} = 0.6,\delta_{T} = 0.3,N_{t} = N_{b} = 0.1 = D = H$$$$,\gamma = 0.7,S_{c} = 0.9,K_{2} = 1$$$$K = E = 1,n = 0.1.$$

$\beta$	$Ha$	$\delta_{v}$	$- C_{f} {\text{Re}}_{x}^{0.5}$
1	0.5	0.3	1.3956622
1.2			1.3101892
1.4			1.2491321
	0.8		1.4442980
	1		1.4773467
	1.2		1.5104225
		0.3	1.3956622
		0.5	1.6751228
		0.7	2.0372521

**Table 3:** Numeric values of $$Nu{\text{Re}}_{x}^{ - 0.5}$$ for distinct values of $$A,\Pr ,K_{1} ,N_{b} ,N_{t}$$ when $$\beta = \omega = Ha = \lambda = \delta_{v} = 0.5,$$$$D = H = 0.1,\delta_{T} = 0.3,\gamma = 0.7,S_{c} = 0.9,K_{2} = 1 = K = E,n = 0.1.$$

$A$	$\Pr$	$K_{1}$	$N_{b}$	$N_{t}$	$Nu{\text{Re}}_{x}^{ - 0.5}$
0.1	2	0.4	0.1	0.1	0.74188429
0.2					0.89769893
0.3					0.96063455
	3				0.74188429
	4				0.78609232
	5				0.82759959
		0.7			0.74696442
		1			0.76313025
		1.2			0.77455931
			0.3		0.67591917
			0.6		0.58303558
			0.9		0.49790646
				0.4	0.72946833
				0.8	0.71232552
				1.2	0.69442108

**Table 4:** Computational values of $Sh{\text{Re}}_{x}^{ - 0.5}$ for several values of $N_{t} ,N_{b} ,K_{2} ,S_{c}$ when $\beta = \omega = Ha = \lambda = \delta_{v} = 0.5,Pr = 2,K_{1} = 0.6D = H = 0.1,\delta_{T} = 0.3,\gamma = 0.7,K = 1 = E,n = 0.1.$

$N_{t}$	$N_{b}$	$K_{2}$	$S_{c}$	$- Sh{\text{Re}}_{x}^{ - 0.5}$
0.4	0.1	0.6	0.9	1.3723983
0.8				1.4531997
1.2				1.5603562
	0.3			1.3249224
	0.6			1.3289993
	0.9			1.3358140
		0.4		1.2902609
		0.7		1.3079720
		1		1.3269492
			1	1.3725206
			1.2	1.4589388
			1.4	1.5399508

## Concluding remarks

The influence of binary chemical reaction and activation energy on a Magnetohydrodynamic dusty Casson nanofluid with modified Fourier and Fick’s laws on a deformable cylinder has numerically been investigated. The flow is analyzed under the impact of variable heat source-sink and Newtonian heating. The formulated mathematical problem is computed by employing bvp4c a built-in function in MATLAB. The salient outcomes of the present exploration are:

- For augmented values of curvature parameter, magnetic parameter and Casson fluid parameter the velocity field diminishes for both the fluid and the dust-particle phase.
- For larger values of momentum dust particle velocity field of the fluid flow declines, whereas, an opposite outcome is noticed for the dust phase.
- By increasing the Newtonian heating the temperature field amplifies for both phases.
- For different values of thermal relaxation time, the temperature field depicts a decreasing behavior for both phases.
- Concentration field deteriorates by increasing $S_{c} ,\delta {\text{ and }}K_{2} .$
- An opposite outcome is observed for $N_{b} {\text{ and }}N_{t}$ on the concentration field.
- By increasing $N_{b} {\text{ and }}N_{t}$ rate of heat transfer reduces.
- The rate of mass transfer amplifies for numerous values of $K_{2} {\text{ and }}S_{c}$.

## Nomenclature

$A = h_{s} \sqrt {\frac{vl}{{u_{0} }}}$: Conjugate parameter for heat transfer
$B_{0}$: Magnetic field strength
$$C$$: Fluid concentration
$$C_{w}$$: Nanoparticle concentration
$$C_{\infty }$$: Ambient concentration
$$c_{p}$$: Specific heat
$c_{m}$: Specific heat of dust particles
$D$: Temperature-dependent source/sink parameter
$D_{b}$: Brownian diffusion coefficient
$D_{t}$: Thermophoretic diffusion coefficient
$$E_{a}$$: Activation energy
$E = \frac{{E_{a} }}{kT}$: Dimensionless activation energy
$H$: Space-dependent source/sink parameter
$$Ha = \frac{{\sigma B_{0}^{2} l}}{{\rho u_{0} }}$$: Magnetic parameter
$h_{s}$: Heat transfer coefficient
$K = 6\pi \mu r$: Stokes’ drag constant
$K_{1} = \frac{{\varepsilon_{T} u_{0} }}{l}$: Thermal relaxation time
$K_{2} = \frac{{\varepsilon_{c} u_{0} }}{l}$: Concentration relaxation time
$$k$$: Thermal conductivity
$$l$$: Characteristic length
$m$: Dust-particle mass
$N$: Number density of the particle phase
$N_{b} = \frac{{\tau D_{b} \left( {C_{w} - C_{\infty } } \right)}}{\nu }$: Brownian motion parameter
$$N_{t} = \frac{{\tau D_{t} }}{\nu }$$: Thermophoresis parameter
$$\Pr = \frac{{\mu c_{p} }}{k}$$: Prandtl number
$$Q_{w}$$: Heat flux
$$Q_{m}$$: Mass flux
$R$: Radius of the cylinder
$Re_{x} = \frac{{x^{2} u_{0} }}{\nu l}$: Local Reynolds number
$$T$$: Fluid temperature
$T_{p}$: Dust particle temperature
$T_{\infty }$: Fluid ambient temperature
$u,w$: Components of velocity
$u_{p} ,w_{p}$: Velocity of dust particles
$$u_{e}$$: Stretching velocity
$$x,r$$: Cylindrical coordinates

## Greek symbols

$$\zeta$$: Similarity variable
$$\sigma_{1}$$: Electrical conductivity
$$\nu$$: Kinematic viscosity
$\rho$: Density of fluid
$\rho_{p} = mN$: Dust-particle density
$$\omega = \left( {\frac{l\nu }{{u_{0} R^{2} }}} \right)^{1/2}$$: Curvature parameter
$\mu_{c}$: Dynamic viscosity of Casson fluid
$\lambda = \frac{Nm}{\rho }$: Mass concentration of dust particle
$\delta_{v} = \frac{l}{{u_{0} \tau_{v} }}$: Fluid particle interaction parameter
$\tau_{v} = \frac{m}{K}$: Dust-particle relaxation time
$\beta = \frac{{u_{B} \left( {2\pi } \right)^{1/2} }}{{S_{y} }}$: Casson fluid parameter
$\tau = \frac{{\left( {\rho c_{p} } \right)}}{{\left( {\rho c_{p} } \right)_{f} }}$: Ratio of specific heat
$$\Lambda^{2}$$: Reaction rate
$\delta = \frac{{\Lambda^{2} l}}{{u_{0} }}$: Reaction rate constant
